# Rapid Focused Sequencing: A Multiplexed Assay for Simultaneous Detection and Strain Typing of *Bacillus anthracis, Francisella tularensis,* and *Yersinia pestis*

**DOI:** 10.1371/journal.pone.0056093

**Published:** 2013-02-13

**Authors:** Rosemary S. Turingan, Hans-Ulrich Thomann, Anna Zolotova, Eugene Tan, Richard F. Selden

**Affiliations:** NetBio, Waltham, Massachusetts, United States of America; University of Louisville, United States of America

## Abstract

**Background:**

The intentional release of *Bacillus anthracis* in the United States in 2001 has heightened concern about the use of pathogenic microorganisms in bioterrorism attacks. Many of the deadliest bacteria, including the Class A Select Agents *Bacillus anthracis, Francisella tularensis,* and *Yersinia pestis,* are highly infectious via the pulmonary route when released in aerosolized form. Hence, rapid, sensitive, and reliable methods for detection of these biothreats and characterization of their potential impact on the exposed population are of critical importance to initiate and support rapid military, public health, and clinical responses.

**Methodology/Principal Findings:**

We have developed microfluidic multiplexed PCR and sequencing assays based on the simultaneous interrogation of three pathogens per assay and ten loci per pathogen. Microfluidic separation of amplified fluorescently labeled fragments generated characteristic electrophoretic signatures for identification of each agent. The three sets of primers allowed significant strain typing and discrimination from non-pathogenic closely-related species and environmental background strains based on amplicon sizes alone. Furthermore, sequencing of the 10 amplicons per pathogen, termed “Rapid Focused Sequencing,” allowed an even greater degree of strain discrimination and, in some cases, can be used to determine virulence. Both amplification and sequencing assays were performed in microfluidic biochips developed for fast thermal cycling and requiring 7 µL per reaction. The 30-plex sequencing assay resulted in genotypic resolution of 84 representative strains belonging to each of the three biothreat species.

**Conclusions/Significance:**

The microfluidic multiplexed assays allowed identification and strain differentiation of the biothreat agents *Bacillus anthracis, Francisella tularensis,* and *Yersinia pestis* and clear discrimination from closely-related species and several environmental background strains. The assays may be extended to detect a large number of pathogens, are applicable to the evaluation of both environmental and clinical samples, and have the potential to be applied in military, public health, and clinical diagnostic settings.

## Introduction

The rapid detection of environmental and clinical biothreat agents will play an increasingly important role in protecting the population, and improved methods of doing so represent an urgent national need [1], [2]. For both clinical and environmental biothreat detection, it would be preferable for a single nucleic acid based assay to interrogate an unprocessed sample for dozens of pathogens. Just as importantly, each pathogen should be interrogated at multiple loci to ensure sensitive and specific detection. Taken together, these two goals suggest the use of highly multiplexed assays designed to interrogate simultaneously hundreds of loci or more. Furthermore, the selection of the loci to be interrogated should allow strain typing to assist in forensic attribution (e.g., geographical origin, subspecies, biovars), to determine virulence and antibiotic resistance status, and to inform initiation of appropriate treatment modalities. In addition, the loci should be chosen to allow discrimination of a given pathogen from non-pathogenic closely-related species (including non-pathogenic near neighbors), environmental background strains (EBS), and common clinical contaminants secondary to sample handling.

Several PCR-based assays have been described for the identification of biothreats [3], [4]. Many such assays rely on single or dual target detection of either chromosomally [5] or plasmid-encoded [6] loci, but these assays may generate false negatives due to presence of strains that have lost their plasmids or near neighbor strains that harbor highly homologous chromosomal loci. A 3-plex PCR assay coupled with microarray-attached probe detection suffers from the same drawbacks as the single and duplex assays, as it too targets single loci specific for each of three biothreat agents [7]. A real time-PCR assays specific for *Yp* targets 6 loci in two separate reactions [8] or 4 loci in one reaction [9]. Furthermore, a recently described 10-plex RT-PCR assay simultaneously targets 3 loci from each of *Ba, Ft,* and *Yp* [10]. Even these multiplexed assays do not allow extensive characterization of the biothreat due to the relatively limited number of probed loci [11], [12]. Microfluidics offers the potential for the development of even more highly multiplexed assays. In particular, the combination of microfluidic amplification and electrophoretic separation and detection is extremely powerful. Labeling amplicons with 4, 6, 8 or more fluorescent dyes in a high-resolution separation system allows a given separation channel to provide hundreds of bases of sequence or to distinguish thousands of amplicons. We have previously developed rapid microfluidic PCR assays that perform simultaneous amplification of up to 27 human loci in under 20 minutes using a reaction volume of 7 µl with near single copy limit of detection [13], [14].

### Molecular Identification of *Bacillus anthracis (Ba)* Strains

*Ba* is considered a highly monomorphic species [15] as it contains few sequence polymorphisms across isolates [16], [17] (greater than 99% sequence identity between strains). The organism harbors an approximately 5.6 Mb chromosome and typically two plasmids, pXO1 (approximately 182 kb) and pXO2 (approximately 96 kb), that are essential for virulence and toxicity. *Ba* is a phylogenetically young species (10–20,000 years old) and closely related to *Bacillus cereus* (*Bc*) and *Bacillus thuringiensis* (*Bt*). The three species are often referred to as *B. cereus senso lato* due to their close phenotypic and genotypic characteristics [18], [19]. Sequence identity within that group is considerable and, accordingly, classic molecular strain typing methods such as rDNA sequence analysis and a conventional Multilocus Sequence Typing (MLST) approach are not suited for identifying and genotyping *Ba* from environmental samples that may also contain other members of the *Bc senso lato* group [20]–[22]. A well-established *Ba* genotyping method is based on VNTR-repeats [23], [24], and a subset of 25 of these loci was recently used to genotype *Ba* isolates by sizing the resulting PCR products via capillary electrophoresis. Although an excellent approach to typing isolated *Ba* strains, VNTR typing is not well-suited to the analysis of environmental samples that may contain other members of the *Bc senso lato* group. Furthermore, this assay requires four separate PCR reactions [25].

### Molecular Identification of *Francisella tularensis (Ft)* Strains

The genus *Francisella* is subdivided into at least three species including, *F. philomiragia* (*Fp*), and other *Francisella* spp. Three of the four recognized *Ft* subspecies, subspp. *tularensis* (also known as *nearctica,* biovar A), *holarctica* (*palearctica*, biovar B), and *mediasiatica* are human pathogens whereas subsp. *novicida* is non-pathogenic to healthy individuals [26]. The North American subsp. *tularensis* causes Type A Tularemia, which has a high mortality rate [27]. In contrast, subsp. *holarctica* and *mediasiatica* are found primarily outside of North America and cause the milder Type B Tularemia. *Fp* and the other *Francisella* spp. are not known to be human pathogens. The conventional MLST approach revealed only moderate sequence type diversity within subspecies (e.g., 3 sequence types in 8 North American *tularensis* isolates) [28], with even lower genotypic resolution obtained by rDNA typing [29], [30]. Genotypic resolution improves with the interrogation of loci harboring gene rearrangements, deletions and insertions, and SNPs [6], [31]. Both Multi Spacer Typing (MST) [32] and Multiple Locus VNTR Analysis (MLVA) [33], [34] are suitable *Ft* genotyping methods. For example, using 25 MVLA loci, 120 genotypes were observed in 192 global *Ft* isolates [35].

### Molecular Identification of *Yersinia pestis (Yp)* Strains

Like *Ba*, *Yp* is considered a genetically monomorphic and phylogenetically young species, having branched from *Y. pseudotuberculosis* (*Ypst*) 1,500–20,000 years ago [36], [37] and with which it shares identical 16S rDNA sequences [38]. Three *Yersinia* species, *Yp, Ypst* and *Y. enterocolitica* (*Ye*) are serious human health threats (*Ypst* and *Ye* are water and food-borne pathogens), while certain other *Yersinia* species are opportunistic pathogens. *Yp* is subdivided into biovars *antiqua* (ant), *mediaevalis* (med) and *orientalis* (ori) that are associated with geographical origin in Africa, Central Asia, and East Asia, respectively. Biovar ori is the only naturally-occurring *Yp* in North America. *Yp* contains a 4.6–4.8 Mbp chromosome and several plasmids of varying size (0.06–0.12 Mb). The most prevalent plasmids are pMT1 (100–110 kb) and pCD1 (70–75 kb). *Yp* strains have been characterized by analysis of VNTRs [39] via MLVA [37], [40] and MST [41]. While conventional MLST analysis is less informative due to high sequence conservation [36], genotyping focusing on heterogeneity in certain metabolism genes can provide valuable information [37], [42].

Taken together, *Ba, Ft*, and *Yp* are representative of biothreats in general in that it is clear that simultaneous interrogation of a large number of loci is optimal for detection and characterization of biothreats. Here, we present two related multiplexed assays. First, we developed a highly multiplexed PCR assay based on the amplification and sizing of ten loci per pathogen, and, in a model system, detected *Ba*, *Ft*, and *Yp* in a single reaction. For all three pathogens, significant strain typing is accomplished by differential fragment sizing of several of the resulting amplicons and discrimination from closely-related species (including non-pathogenic near neighbors) and EBS based on selective primer binding. Furthermore, we show that the assay can provide an initial characterization of the detected agent including geographical origin, subspecies, and biovar. The amplification of 10 target loci from each of the three pathogens achieves specificity and genotypic resolution based on the resulting Fragment Length Type (FLT) obtained by microfluidic electrophoretic fragment size analysis. Second, we extended the amplification assay by performing microfluidic Sanger sequencing on the amplicons. The Rapid Focused Sequencing of approximately ten loci per pathogen allows an even greater degree of strain typing than PCR alone. Both the highly multiplexed fragment sizing and sequencing assays hold promise to support a rapid, field-forward, and self-verifying rapid response in cases of suspected release of *Ba, Ft* or *Yp*, and ultimately, a broad range of biothreat pathogens.

## Results

**Figure 1:**
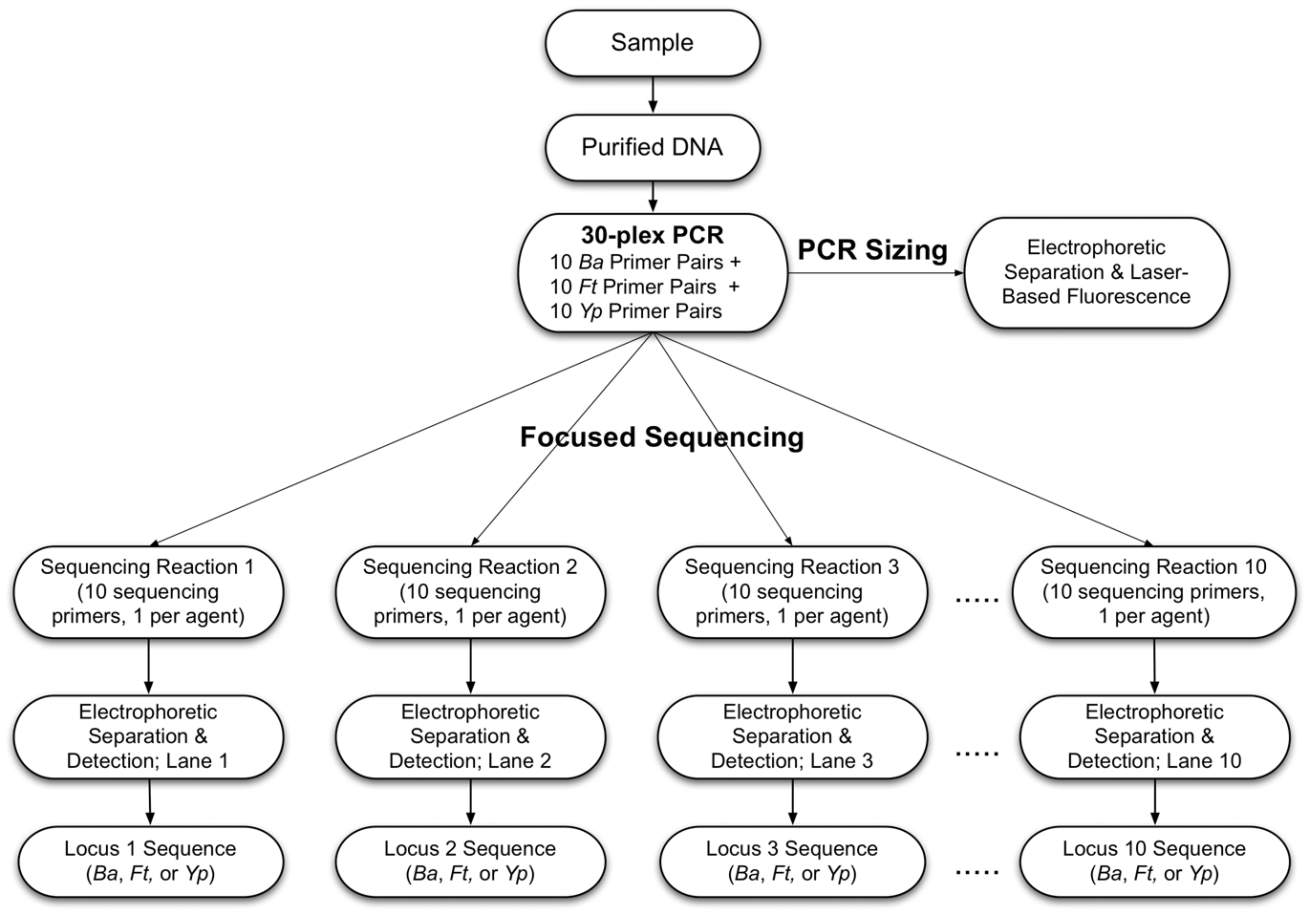
Flowchart illustrating process steps of the two related multiplexed assays: PCR Sizing and Rapid Focused Sequencing. In the multiplexed PCR sizing assay, the PCR reaction products are separated by microfluidic electrophoresis and detected based on labeled primers incorporated during the amplification reaction. In the Rapid Focused Sequencing assay, the multiplexed amplification utilizes the identical but unlabeled 30-plex primer set. The resulting PCR reaction products are then subjected to Sanger Sequencing followed by microfluidic electrophoresis and detection based on labeled primers incorporated during the sequencing reaction.

An overview of the two closely-related multiplexed assays is presented in Figure 1. In both cases, purified nucleic acids are subjected to a multiplexed (in this case 30-plex) microfluidic amplification in a 7 µl reaction volume. In the multiplexed PCR sizing assay, the PCR reaction products are separated by microfluidic electrophoresis and detected based on labeled primers incorporated during the amplification reaction. In the Focused Sequencing assay, the multiplexed amplification utilizes the identical but unlabeled 30-plex primer set. Following amplification, the reaction is spit into 20 equal aliquots, and each aliquot is diluted to for microfluidic Sanger sequencing. Each sequencing reaction contains a forward or reverse sequencing primer for one locus from each of the three pathogens. The 20 reactions therefore comprise one forward and one reverse sequencing primer for all ten loci for the three agents, and each sequencing reaction is separated and detected on an individual electrophoresis channel.

Development of the two assays for detection and characterization of the biothreat agents *Ba, Ft* and *Yp* was conducted in five steps: (1) selecting targets and designing primers using available whole genome sequence (WGS) data of the biothreat and their near neighbor species; (2) testing of primer specificity and sensitivity in singleplex PCR with reference strain template DNAs representing each agent; (3) combining 10 primer pairs specific for each agent into a 10-plex PCR panel, followed by iterative primer testing and redesign to ensure all loci were capable of simultaneous amplification; (4) testing specificity and sensitivity of each 10-plex panel, including sequence analysis of all amplicons; and (5) combining the three 10-plex panels to generate a 30-plex PCR panel for simultaneous detection and characterization of all three biothreat agents, followed by sequence analysis of all amplicons.

### *B. anthracis* Multiplexed PCR Sizing and Rapid Focused Sequencing Assays

Using published WGS data from 19 *Ba* strains and from *Ba* near neighbor species in the *B. cereus sensu lato* group, we evaluated a number of loci for inclusion in the multiplex panel. Potential targets were screened to fulfill at least two of the following criteria: (1) highly conserved in *Ba*, i.e., present in all *Ba* strain WGS data (except for plasmid encoded loci); (2) species-specific, i.e., sequence variations in the target between *Ba* and its near neighbors can be utilized for designing *Ba* specific primers; and (3) discriminating, i.e., motifs within the target are useful for *Ba* genotyping and discrimination of *Ba* against near neighbors. Note that interrogating *Ba* plasmid loci is important to identify strains harboring toxin and virulence genes but is insufficient to unambiguously identify *Ba* within the *Bc senso lato* group. Certain *Bacillus* species have been shown to harbor sequences highly homologous or identical to pXO1 and pXO2-encoded genes [43], [44]. For example, *B. cereus* strain G9241 harbors pBCXO1, a pXO1-like plasmid [45] and *B. cereus* biovar *anthracis* CI contains two plasmids with very high sequence identity as compared to pXO1 and pXO2 [46]. However, those isolates are less closely related to *Ba* based on chromosomal sequence identity. Accordingly, we also selected the *Ba* genotyping targets to balance chromosomal and plasmid-specific information to distinguish *Ba* from pXO-harboring near neighbors from non-*Ba* species and from attenuated or avirulent *Ba* strains that have lost one or both plasmids [47]–[50].

Eight chromosomally-encoded and two pXO1-encoded loci were selected for primer pair design for inclusion in the 10-plex PCR *Ba* panel. They include parts of genes encoding conserved sporulation protein and sporulation regulation factors (*ssp*F and *spo*VT, respectively), a housekeeping enzyme (*hem*L), a metabolism enzyme (GBAA0872), conserved plasmid-encoded virulence factors and toxins (*lef* and *ger*XB), and loci harboring VNTR-like sequences (*bas*B and *pbp*1A) and non-coding spacer regions (adjacent to *yih*Y and *cod*Y). Development, assembly, and optimization of the multiplexed panel were performed and amplification and initial sequence data generated prior to the acquisition of the necessary permit to obtain and handle non-attenuated genomic DNAs from several pXO2-harboring agents. Hence, primers for the pXO2-encoded target were not included in the final assembly but will be incorporated into an expanded panel in the future.

To enable laser-induced detection of resulting amplicons from each locus, one primer from each pair was labeled at the 5′ end with one of the fluorescent dyes FAM, JOE, TMR or ROX. The labels were selected to allow appropriate spectral resolution of the PCR products based on their predicted fragment sizes. Primer sequences and target information are shown in Table S1. Primer pairs were initially tested in rapid singleplex PCR reactions followed by fragment size analysis on Genebench, a microfluidic electrophoresis instrument, as described in Material and Methods. PCR specificity, as judged by presence of a PCR product of the expected length, and PCR sensitivity, as judged by fluorescent signal strength, were evaluated in the presence of 50 genome equivalents of the pXO2-deficient Sterne_2 strain DNA. Successful primer pairs were then combined sequentially to generate the 10-plex PCR *Ba* panel. The panel was tested in reactions containing template DNA from 20 *Ba* strains and 10 *Ba* near neighbors, including *B. cereus* (*Bc*) and *B. thuringiensis* (*Bt*) strains.

**Figure 2:**
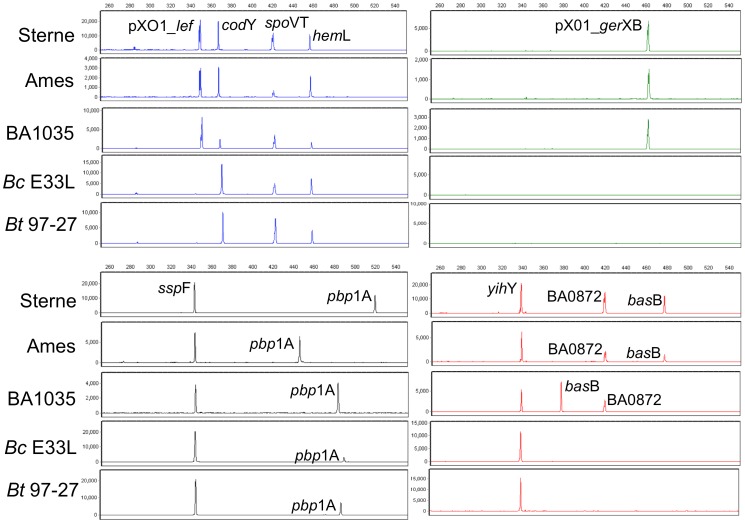
10-plex PCR profiles generated from representative *Ba* and *Ba*NN strains. As input to PCR, 100 copies of template *Ba* strains Sterne, Ames, and BA1035; and 1000 copies of template *Ba*NN strains *Bc* E33L and *Bt* 97-27, were used. The FAM (blue), JOE (green), TMR (black) and ROX (red) labeled products are aligned to illustrate amplicon sizes from the set of five strains. As expected, all 10 loci were amplified in the 3 *Ba* strains; only 6 of 10 loci were observed in *Ba*NNs. The *bas*B and *pbp*1A amplicons allow discrimination between strains Ames, Sterne, and BA1035; *pbp*1A fragment length also distinguishes *Bc* E33L from *Bt* 97-27. X-axes show fragment size in base pairs and Y-axes relative fluorescence units (rfu).

The multiplexed PCR sizing assay allows discrimination of *Ba* strains and near neighbors. Figure 2 shows the resulting electropherograms from *Ba* strains Sterne, Ames, and BA1035, from which all *Ba* 10 loci are amplified, and the closely-related species *Bc* E33L and *Bt* 97-27, from which only 6 out of 10 loci are amplified. *Bc* E33L and *Bt* 97-27 are missing the two pXO1 amplicons (*lef,* gerXB) because they do not harbor the plasmid, the BA0872 amplicon due to the absence of the gene, and the *bas*B-amplicon due to primer/target mismatches, respectively. Eight amplicons (*ssp*F, *spo*VT, BA0872, *hem*L, *yih*Y, *cod*Y, *lef* and *ger*XB) do not vary in length among the *Ba* examples, but the presence and length of these amplicons unambiguously indicates the presence of *Ba* DNA. The observed Fragment Length Types (FLTs, defined as the amplicon fragment lengths observed for a given locus) in the *bas*B and *pbp*1A amplicons allow discrimination between strains Ames, Sterne, and BA1035; fragment length in *pbp*1A also distinguishes *Bc* E33L from *Bt* 97-27. In addition, the FLTs of *pbp*1A in *Bc* E33L and *Bt* 97-27 are not present in any of the *Ba* strains analyzed herein.

The experimentally determined and *in silico* predicted FLT from the 10 loci generated from 36 *Ba* strains and 12 *Ba* near neighbor species strains are listed in Table 1. As predicted *in silico*, PCR products from loci harboring VNTR-like repeats and indels (e.g., *bas*B and *pbp*1A), display the highest degree of fragment size variation among *Ba* strains. Fragments specific for the other 8 loci vary to a lesser degree, generally by 1–2 bp between strains. A total of 20 high resolution amplified fragment length polymorphisms (hAFLP) types (defined as the combined FLTs of 10 loci) were detected in 36 *Ba* strains (Table 1; the 20 hAFLP types include 5 WGS strains that have no homology for one or two housekeeping genes (most likely due to physical sequence gaps). Several of these strains are expected to share identical hAFLP types as they are closely related or identical. For example, the closely related Ames, Ames 35, and A2012 strains share hAFLP type a, and all Sterne and Sterne strain derivatives share hAFLP type e.

**Table 1:** hAFLP types and Sequence Types (STs) of *Ba* strain and *Ba* near neighbor species DNA determined experimentally (shown in bold) and by *in silico* analysis of WGS strains (shown in italics).

Strain [Genbank Acc#]	hAFLP	ST	*ssp*F		*spo*VT		BA0872		*hem*L		*bas*B		*pbp*1A		*yih*Y		*cod*Y		pXO1_*lef*		XO1_*ger*XB	
*Bacillus anthracis*			FLT	GT	FLT	GT	FLT	GT	FLT	GT	FLT	GT	FLT	GT	FLT	GT	FLT	GT	FLT	GT	FLT	GT
**Ames [AE017334.2]**	**a**	**A**	**1**	**1**	**1**	**1**	**1**	**1**	**1**	**1**	**1**	**1**	**1**	**1**	**1**	**1**	**1**	**1**	**1**	**1**	**1**	**1**
**Ames 35**	**a**	**A**	**1**	**1**	**1**	**1**	**1**	**1**	**1**	**1**	**1**	**1**	**1**	**1**	**1**	**1**	**1**	**1**	**1**	**1**	**1**	**1**
*Ames Porton [AE016879.1]*	aa	AA	1	1	1	1	1	1	1	1	1	1	1	1	1	1	1	1	Δ	Δ	Δ	Δ
*A2012 [AAAC000000]*	a*	A*	1	1	1	1	1	1	#	#	1	1	1	1	1	1	#	#	1	1	1	1
*A0248 [CP001598.1]*	a	A	1	1	1	1	1	1	1	1	1	1	1	1	1	1	1	1	1	1	1	1
**Vollum 1B [AAEP000000]**	**b**	**B**	**1**	**1**	**1**	**1**	**1**	**1**	**1**	**1**	**2**	**2**	**2**	**2**	**1**	**1**	**1**	**1**	**1**	**1**	**1**	**1**
*A0488 [ABJC000000*]	b*	B*	1	1	1	1	1	1	1	1	2	2	2	2	1	1	#	#	1	1	1	1
**RA3**	**d**	**D**	**1**	**1**	**1**	**1**	**1**	**1**	**1**	**1**	**3**	**3**	**3**	**3**	**1**	**1**	**2**	**2**	**1**	**3**	**1**	**1**
*A0465 [ABLH000000]*	d*	D*	1	1	1	1	1	1	1	1	3	3	3	3	1	1	#	#	1	3	1	1
*CNEVA-9066 [AAEN000000]*	d	D	1	1	1	1	1	1	1	1	3	3	3	3	1	1	2	2	1	3	1	1
**Zimbabwe 89**	**c**	**C**	**1**	**1**	**1**	**2**	**1**	**1**	**1**	**1**	**4**	**4**	**4**	**4**	**1**	**1**	**1**	**1**	**1**	**3**	**1**	**1**
*A0442 [ABKG000000]*	c*	C*	1	1	1	2	1	1	1	1	4	4	4	4	1	1	#	#	1	3	1	1
*Kruger B [AAEQ000000]*	c	C	1	1	1	2	1	1	1	1	4	4	4	4	1	1	1	1	1	3	1	1
**BA1035**	**c**	**C**	**1**	**1**	**1**	**2**	**1**	**1**	**1**	**1**	**4**	**4**	**4**	**4**	**1**	**1**	**1**	**1**	**1**	**3**	**1**	**1**
**Sterne [AE017225.1]**	**e**	**E**	**1**	**1**	**1**	**1**	**1**	**1**	**1**	**1**	**1**	**1**	**2**	**2**	**1**	**1**	**1**	**1**	**1**	**2**	**1**	**1**
**Sterne**	**e**	**E**	**1**	**1**	**1**	**1**	**1**	**1**	**1**	**1**	**1**	**1**	**2**	**2**	**1**	**1**	**1**	**1**	**1**	**2**	**1**	**1**
**Sterne**	**e**	**E**	**1**	**1**	**1**	**1**	**1**	**1**	**1**	**1**	**1**	**1**	**2**	**2**	**1**	**1**	**1**	**1**	**1**	**2**	**1**	**1**
**Weybridge**	**e**	**E**	**1**	**1**	**1**	**1**	**1**	**1**	**1**	**1**	**1**	**1**	**2**	**2**	**1**	**1**	**1**	**1**	**1**	**2**	**1**	**1**
**Sterne_2**	**e**	**E**	**1**	**1**	**1**	**1**	**1**	**1**	**1**	**1**	**1**	**1**	**2**	**2**	**1**	**1**	**1**	**1**	**1**	**2**	**1**	**1**
**Pasteur**	**m**	**M**	**2**	**2**	**1**	**1**	**1**	**1**	**1**	**1**	**2**	**2**	**2**	**2**	**1**	**1**	**1**	**1**	**n**	**n**	**n**	**n**
*CDC 684 [CP001215.1]*	**f**	**F**	**1**	**1**	**1**	**1**	**1**	**1**	**1**	**1**	**1**	**7**	**5**	**5**	**2**	**2**	**1**	**1**	**1**	**1**	**1**	**1**
**Canadian Bison**	**l**	**L**	**1**	**1**	**1**	**1**	**1**	**1**	**1**	**1**	**6**	**6**	**2**	**2**	**1**	**1**	**1**	**1**	**1**	**1**	**1**	**1**
**V770-NP-1R**	**l**	**L**	**1**	**1**	**1**	**1**	**1**	**1**	**1**	**1**	**6**	**6**	**2**	**2**	**1**	**1**	**1**	**1**	**1**	**1**	**1**	**1**
**A0370**	**e**	**E**	**1**	**1**	**1**	**1**	**1**	**1**	**1**	**1**	**1**	**1**	**2**	**2**	**1**	**1**	**1**	**1**	**1**	**2**	**1**	**1**
*USA6153 [AAER000000]*	g	G	1	1	1	1	1	1	1	1	2	2	2	2	1	1	2	3	1	1	1	1
*A0174 [ABLT000000]*	g*	G*	1	1	1	1	1	1	1	1	2	2	2	2	1	1	#	#	1	1	1	1
*A0193 [ABKF000000]*	l*	L*	1	1	1	1	1	1	1	1	6	8	2	2	1	1	#	#	1	1	1	1
**2002013094**	**n**	**N**	**1**	**1**	**1**	**1**	**1**	**1**	**1**	**1**	**5**	**5**	**2**	**7**	**1**	**1**	**2**	**4**	**1**	**4**	**1**	**2**
**K1811**	**e**	**O**	**1**	**1**	**1**	**1**	**1**	**1**	**1**	**1**	**1**	**1**	**2**	**2**	**1**	**1**	**1**	**1**	**1**	**1**	**1**	**1**
*Australia 94 [AAES000000]*	e	O	1	1	1	1	1	1	1	1	1	1	2	2	1	1	1	1	1	1	1	1
A1055 [AAEO000000]	j	J	1	1	1	1	1	1	1	1	5	5	2	7	2	2	2	2	Δ	Δ	Δ	Δ
**SK-102**	**e**	**P**	**1**	**1**	**1**	**1**	**1**	**1**	**1**	**2**	**1**	**1**	**2**	**2**	**1**	**1**	**1**	**1**	**1**	**1**	**1**	**1**
**PAK-1**	**b**	**B**	**1**	**1**	**1**	**1**	**1**	**1**	**1**	**1**	**2**	**2**	**2**	**2**	**1**	**1**	**1**	**1**	**1**	**1**	**1**	**1**
**Turkey 32**	**b**	**B**	**1**	**1**	**1**	**1**	**1**	**1**	**1**	**1**	**2**	**2**	**2**	**2**	**1**	**1**	**1**	**1**	**1**	**1**	**1**	**1**
*Tsiankovskii-I [ABDN000000]*	i*	I*	1	1	1	1	1	1	1	1	2	2	4	8	1	1	#	#	1	1	1	1
*A0389 [ABLB000000]*	h*	H*	1	1	1	1	1	1	1	1	1	1	4	6	1	1	#	#	1	1	1	1

Near Neighbor strains:			FLT	GT	FLT	GT	FLT	GT	FLT	GT	FLT	GT	FLT	GT	FLT	GT	FLT	GT	FLT	GT	FLT	GT
*Bcbv. anthracis CI [CP001746.1]*	Na	NA	1	1	1	8	1	2	1	3	**n**	**n**	6	11	1	4	3	5	1	6	1	2
***Bc*** **G9241 [AAEK000000]**	Nb	NB	**1**	**3**	**1**	**5**	**n**	**n**	**1**	**7**	**8**	**11**	**6**	**15**	**2**	**6**	**1**	**9**	**1**	**7**	**n**	**n**
***Bc*** **E33L [CP000001.1]**	Nc	NC	**1**	**3**	**1**	**3**	**n**	**n**	**1**	**4**	**n**	**n**	**7**	**14**	**1**	**7**	**3**	**7**	**n**	**n**	**n**	**n**
***Bc*** **3A**	Nd	ND	**1**	**1**	**1**	**1**	**n**	**n**	**1**	**1**	**5**	**9**	**6**	**9**	**1**	**10**	**2**	**6**	**n**	**n**	**n**	**n**
***Bc*** **4342 [ACLZ000000]**	Ne	NE	**1**	**4**	**1**	**7**	**n**	**n**	**1**	**9**	**7**	**10**	**7**	**13**	**2**	**9**	**5**	**10**	**n**	**n**	**n**	**n**
*Bc03BB102 [CP001407.1]*	Nf	NF	1	1	1	6	1	3	1	5	**n**	**n**	6	10	2	5	3	13	1	5	Δ	?
***Bt*** **Al Hakam [CP000485.1]**	Ng	NG	**1**	**1**	**1**	**6**	**n**	**n**	**1**	**5**	**n**	**n**	**6**	**10**	**2**	**5**	**4**	**8**	**n**	**n**	**n**	**n**
***Bt*** **sv. ** ***konkukian*** ** 97-27 [AE017355.1]**	Nh	NH	**1**	**1**	**1**	**4**	**n**	**n**	**1**	**6**	**n**	**n**	**6**	**12**	**1**	**3**	**3**	**5**	**n**	**n**	**n**	**n**
***Bt*** **sv. ** ***kurstaki*** ** HD1**	Ni	NI	**n**	**n**	**n**	**n**	**n**	**n**	**1**	**8**	**n**	**n**	**n**	**n**	**1**	**8**	**5**	**12**	**n**	**n**	**n**	**n**
***Bt*** **sv. ** ***israelensis*** ** 35646 **	Nj	NJ	**n**	**n**	**n**	**n**	**n**	**n**	**n**	**n**	**n**	**n**	**n**	**n**	**n**	**n**	**6**	**11**	**n**	**n**	**n**	**N**
***B. megaterium***	–	–	**n**	**n**	**n**	**n**	**n**	**n**	**n**	**n**	**n**	**n**	**n**	**n**	**n**	**n**	**n**	**n**	**n**	**n**	**n**	**N**
***B. coagulans*** ** ATCC 7050**	–	–	**n**	**n**	**n**	**n**	**n**	**n**	**n**	**n**	**n**	**n**	**n**	**n**	**n**	**n**	**n**	**n**	**n**	**n**	**n**	**N**

Corresponding Genbank accession numbers are in brackets. FLT Fragment Length Type; GT: sequence genotype of locus;

* hAFLP Type and ST assigned despite missing WGS data;

# deletion or physical gap in WGS data; Δ: strain deficient of pXO1 plasmid or locus; n: no PCR product or sequence detected.

Specificity of the *Ba* panel was tested on template DNA from 10 near neighbor strains. hAFLP types recorded from the closely-related species are clearly distinguishable from those observed in the *Ba* group. The distinct fragment sizing profile between *Ba* and near neighbors *B. cereus* E33L and *Bt* 97-27 are also shown in Figure 2. Although 4 of 10 amplicons (*ssp*F, *spo*VT, *hem*L and *yih*Y) share FLTs with *Ba* group strains, the overall pattern is distinct from any observed in *Ba.* BA0872-specific products are missing from the profile of all closely-related species due to the absence of BA0872-specific primer binding sites, and *bas*B-specific fragments are also absent (with the exception of *Bc* G9241, *Bc* 3A, and *Bc* 4342, all of which show discriminatory *bas*B amplicon sizes). As expected, most near neighbors do not yield detectable pXO1-*lef* and pXO1-*ger*XB specific products; an exception is G9241, known to harbor pXO-like plasmids [45]. Three *Ba* isolates, Pasteur (pXO1^−^), A1055, and Ames Porton Down (pXO1^−^, pXO2^−^), do not generate pXO1-*lef* and pXO1-*ger*XB -specific amplicons, as is the case for the majority of the near neighbor strains. However, presence of a BA0872-specific fragment identifies them as likely *Ba* group strains. Conversely, the BA0872 fragment is absent in the profile of near neighbor strain *Bc* 3A, the isolate that shares the greatest similarity to *Ba* based on individual FLTs. Based on hAFLP types, this isolate could represent a plasmid-deficient *Ba* strain with a mutation in the BA872 primer-binding site; sequence data unambiguously eliminates this possibility (see below).

Focused sequencing of the 10 *Ba* panel loci (Table 1) showed a total of 22 Sequence Types (STs, defined as the combined locus sequence genotypes of 10 loci) in the 36 *Ba* isolates. This increase (as compared to 20 hAFLP types) is due to both SNPs and size-neutral indels (insertion/deletions which cancel impact on size) that are detected and by sequencing. For example, strains Australia 94, K1811 and SK-102 are indistinguishable from the Sterne strains and A0370 by fragment sizing. In contrast, sequence analysis revealed SNPs in the pXO1-*lef* and *hem*L amplicons. The contribution of SNPs to the number of observed genotypes is most prevalent in loci pXO1-*lef* (4 GTs versus 1 hAFLP type within *Ba*) and *bas*B (8 GTs versus 6 hAFLP types). In certain cases, the additional genotypic variability observed by Rapid Focused Sequencing does not increase the number of STs but adds to the overall genotyping confidence.

The additional genotypic information gained by Rapid Focused Sequencing as compared to PCR sizing is valuable to clearly distinguish ‘atypical’ *Ba* strains, such as plasmid-deficient strains Pasteur, Ames Porton Down (*in silico* data), and A1055 (*in silico* data) from close near neighbors such as *Bc* 3A (no plasmid loci but almost identical FLTs of chromosomal loci) and *Bc* bv. *anthracis* CI (plasmid loci and chromosomal loci almost identical to *Ba* strains). Every *Ba* near neighbor strain is associated with STs that are distinct from those observed in *Ba* strains, and most closely-related species have unique sequences not observed in any of the *Ba* strains. *B. coagulans* and *B. megaterium* strains did not generate amplicons from any of the 10 loci, an expected outcome as the targets were selected to discriminate against such phylogenetically more distant species. Finally, there were no discrepancies observed between published WGS data of the analyzed near neighbor strains (i.e., *Bt konkukian* 97-27, *Bt* Al Hakam, *Bt kurstaki* HD1, *Bt* sv. *israelensis* ATCC 35646, *Bc* E33L, *Bc* G9241, *Bc* 4342) and the sequence data generated using the *Ba* primer set. Figure S1 shows the phylogenetic tree based solely on the *Ba* 10-plex panel experimentally obtained gene sequences and whole genome sequence available data that have been concatenated after clustal alignment and visualization with PHYLIP (TreeView, [51]). The high conservation within the *Ba* species can be seen; the closely-related *Bc* and *Bt* are clearly distinct from the *Ba* species cluster.

### *F. tularensis* Multiplexed PCR Sizing and Rapid Focused Sequencing Assays

Using published WGS data from 23 *Ft* and *Ft* near neighbors, a total of 10 target loci were selected for primer design (Table S2). Targets were chosen to distinguish 1) the four *Ft* subspecies; and 2) strains within each subspecies, including two highly virulent North American subspecies (*tularensis* type A1 and A2 clade strains). Primers were designed to be discriminatory against homologous *Fp* targets. The *Ft* target panel includes amplicons from virulence (*acp*A, *pdp*D, *igl*C), antigen (*fop*A), putative metabolic (*gyr*, *spe*A), and transcriptional regulator (*mig*R, FTT008) genes. Two amplicons also harbor VNTR-like repeats, indels, and SNPs present within intergenic regions (i.e., between virulence gene *tul*4 and ORF FTT0900, and downstream of ORF FTT0082). Targets FTT0082 and *pep*O are annotated as pseudogenes in certain subspecies (including reference strain Schu S4). These were included in the panel because they are present in all *Ft* subspecies, are highly conserved, and contain variable regions valuable for genotypic resolution. Finally, the *pdp*D gene, encoded on the *Francisella* Pathogenicity Island, is present in type A (subsp. *tularensis*) and most *novicida* strains but deleted in type B (subsp. *holarctica*) strains. One primer from each locus was fluorescently labeled, and primer sequences are found in Table S2.

**Figure 3:**
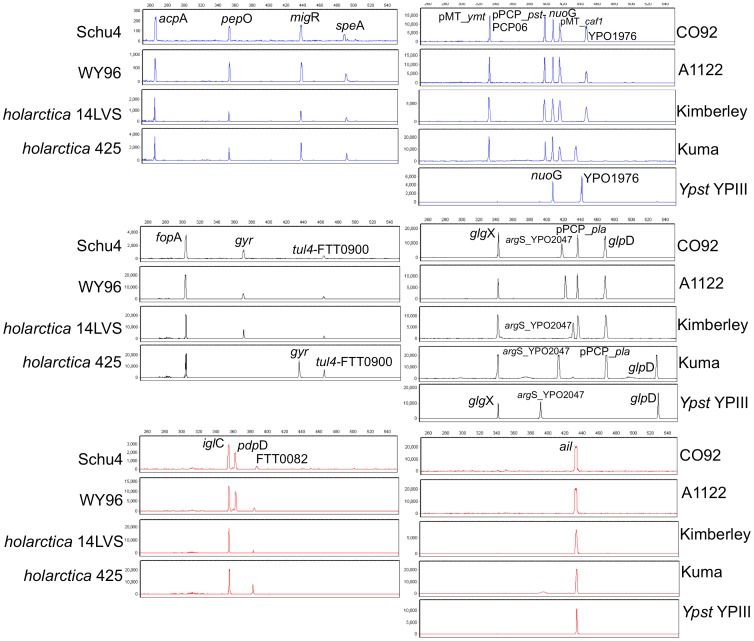
10-plex PCR profiles generated from representative *Ft* strains (left panel) and *Yp* and *Yp*NN strains (right panel). The FAM (blue), TMR (black) and ROX (red) labeled products are aligned to emphasize the variant and non-variant fragment sizes observed across these strains. Six of ten fragments (*acp*A, *pep*O, *mig*R, *fop*A, *tul*4-FTT0900, and *igl*C) in the 10-plex *Ft* panel are invariable in length between strains subsp. *tularensis* SchuS4 and WY96, and subsp. *holarctica* 14LVS and 425. In *Yp* 10-plex panel, size variability is observed primarily in 4 loci (*glp*D, *ail, arg*S-YPO2047 and YPO1976). Both *Ft* 10-plex and *Yp* 10-plex panels do not have any JOE-labeled (green) primers. For each reaction, 100 genome equivalents of each strain were used as template to PCR.

Following individual primer pair optimization and testing, the primer pairs were combined sequentially to generate the 10-plex PCR *Ft* panel. The panel was tested in PCR reactions containing DNA from 100 genome equivalents of *Ft* subsp. *tularensis* Schu S4 DNA and 28 additional *Francisella* strains. Electropherograms obtained from 4 subsp. *tularensis* and *holarctica* strains are shown in Figure 3 (left panel).

Six of the 10 fragments in the 10-plex *Ft* panel are invariable in length between strains subsp. *tularensis* SchuS4 and WY96, and subsp. *holarctica* 14LVS and 425. The lack of *pdp*D-specific product in the profiles of the two *holarctica* strains is expected as this locus is deleted in that subspecies but not in subsp. *tularensis* and *novicida* strains. In contrast, the VNTR-like repeat containing the *gyr*-specific amplicon of strain 425 differs significantly in length as compared to those from SchuS4, WY96 and 14LVS (Figure 3). Five distinct *gyr* FLTs were observed in the analyzed strains. In contrast, the PCR products of *spe*A and FT0082 differ by only 1 or 2 bp across strains. However, as these two loci have several indel regions that also result in neutral size variation (one indel cancels the other), sequence analysis is better suited to discriminate between isolates. VNTR-like repeat motifs in the *Ft* panel loci (e.g., *gyr,* speA), exhibit less fragment size variation than that observed in VNTR loci of the *Ba* and *Yp 10-plex* panels. Nevertheless, size variability still allows classification of all examined strains into species and subspecies.

Experimentally determined and *in silico* predicted individual FLTs and combined hAFLP types from 31 *Ft* strains and 13 *Ft* near neighbor species strains are listed in Table 2. Sixteen hAFLP types were observed; three hAFLP types segregate with the highly virulent types A1 (hAFLP types a and b) and A2 (hAFLP type c), while seven hAFLP types (d, e, f, g, h, n, o) are associated with less virulent type B strains. Failure to obtain detectable *pdp*D-specific PCR products in type B strains is expected as all previously characterized *holarctica* strains carry a *pdp*D deletion [52]. Non-pathogenic near neighbor *Ft* subsp. *novicida* strains have distinct hAFLP types (hAFLP types k, j, m, l, p) as compared to the pathogenic *tularensis* subtypes, and the three analyzed *Fp* strains fail to amplify all but one locus, leading to unambiguous distinction from pathogenic *Ft* subspp. Although predicted by *in silico* analysis, non-pathogenic near neighbor strain *novicida* GA99-3548 did not yield a FTT0082 specific fragment in the 10-plex PCR, and the *novicida* strain GA99-3549 yielded no product from the *pdp*D gene. This discrepancy between published WGS and experimental data could be due to a mutation that affects one or both primer-binding sites in the here-analyzed isolate as compared to the WGS strain.

**Table 2:** hAFLP types and Sequence Types (STs) of *Ft* strains and *Ft* near neighbor species DNA (shown in bold) and by *in silico* analysis of WGS (shown in italics).

Strain [Genbank Acc#]	hAFLP	ST	*igl*C		*fop*A		*tul*4-FT900		*pdp*D		*acp*A		*pep*O		*mig*R		*gyr*		*spe*A		FT82	
subspecies *tularensis*			FLT	GT	FLT	GT	FLT	GT	FLT	GT	FLT	GT	FLT	GT	FLT	GT	FLT	GT	FLT	GT	FLT	GT
**Schu S4 [AJ749949.2]**	**a**	**A**	**1**	**1**	**1**	**1**	**1**	**1**	**1**	**1**	**1**	**1**	**1**	**1**	**1**	**1**	**1**	**1**	**1**	**1**	**1**	**1**
*FSC198 [AM286280.1]*	a	A	1	1	1	1	1	1	1	1	1	1	1	1	1	1	1	1	1	1	1	1
*FSC033 [AAYE00000]*	a	A	1	1	1	1	1	1	1	1	1	1	1	1	1	1	1	1	1	1	1	1
**MA00-2987 [ABRI00000]**	**a**	**A**	**1**	**1**	**1**	**1**	**1**	**1**	**1**	**1**	**1**	**1**	**1**	**1**	**1**	**1**	**1**	**1**	**1**	**1**	**1**	**1**
*NE061598 [CP001633]*	c	C	1	1	1	1	1	1	1	1	1	1	1	1	1	1	2	2	1	1	1	1
**Scherm**	**c**	**C**	**1**	**1**	**1**	**1**	**1**	**1**	**1**	**1**	**1**	**1**	**1**	**1**	**1**	**1**	**2**	**2**	**1**	**1**	**1**	**1**
***tularensis*** ** FRAN005**	**c**	**C**	**1**	**1**	**1**	**1**	**1**	**1**	**1**	**1**	**1**	**1**	**1**	**1**	**1**	**1**	**2**	**2**	**1**	**1**	**1**	**1**
***tularensis*** ** FRAN006**	**c**	**C**	**1**	**1**	**1**	**1**	**1**	**1**	**1**	**1**	**1**	**1**	**1**	**1**	**1**	**1**	**2**	**2**	**1**	**1**	**1**	**1**
***tularensis*** ** FRAN007**	**c**	**C**	**1**	**1**	**1**	**1**	**1**	**1**	**1**	**1**	**1**	**1**	**1**	**1**	**1**	**1**	**2**	**2**	**1**	**1**	**1**	**1**
***tularensis*** ** FRAN008**	**c**	**C**	**1**	**1**	**1**	**1**	**1**	**1**	**1**	**1**	**1**	**1**	**1**	**1**	**1**	**1**	**2**	**2**	**1**	**1**	**1**	**1**
***tularensis*** ** FRAN009**	**c**	**C**	**1**	**1**	**1**	**1**	**1**	**1**	**1**	**1**	**1**	**1**	**1**	**1**	**1**	**1**	**2**	**2**	**1**	**1**	**1**	**1**
***tularensis*** ** FRAN011**	**c**	**C**	**1**	**1**	**1**	**1**	**1**	**1**	**1**	**1**	**1**	**1**	**1**	**1**	**1**	**1**	**2**	**2**	**1**	**1**	**1**	**1**
***tularensis*** ** FRAN015**	**c**	**C**	**1**	**1**	**1**	**1**	**1**	**1**	**1**	**1**	**1**	**1**	**1**	**1**	**1**	**1**	**2**	**2**	**1**	**1**	**1**	**1**
**WY96 [CP000608.1]**	**b**	**B**	**1**	**2**	**1**	**3**	**1**	**2**	**1**	**2**	**1**	**1**	**1**	**2**	**1**	**1**	**1**	**1**	**1**	**2**	**2**	**2**
**ATCC 6223**	**B**	**B**	**1**	**2**	**1**	**3**	**1**	**2**	**1**	**2**	**1**	**1**	**1**	**2**	**1**	**1**	**1**	**1**	**1**	**2**	**2**	**2**
**subspecies ** ***holarctica***			**FLT**	**GT**	**FLT**	**GT**	**FLT**	**GT**	**FLT**	**GT**	**FLT**	**GT**	**FLT**	**GT**	**FLT**	**GT**	**FLT**	**GT**	**FLT**	**GT**	**FLT**	**GT**
**LVS [AM233362.1]**	**d**	**D**	**1**	**2**	**1**	**2**	**1**	**3**	**n**	**n**	**1**	**2**	**1**	**1**	**1**	**2**	**1**	**1**	**2**	**3**	**3**	**3**
**14LVS**	**d**	**D**	**1**	**2**	**1**	**2**	**1**	**3**	**n**	**n**	**1**	**2**	**1**	**1**	**1**	**2**	**1**	**1**	**2**	**3**	**3**	**3**
**LVSG**	**d**	**D**	**1**	**2**	**1**	**2**	**1**	**3**	**n**	**n**	**1**	**2**	**1**	**1**	**1**	**2**	**1**	**1**	**2**	**3**	**3**	**3**
**JAP**	**d**	**L**	**1**	**2**	**1**	**2**	**1**	**3**	**n**	**n**	**1**	**2**	**1**	**1**	**1**	**1**	**1**	**1**	**2**	**4**	**3**	**3**
*FSC257 [AAUD00000]*	**d**	F	1	3	1	2	3	4	Δ	Δ	1	2	1	1	1	2	1	1	2	3	3	3
**VT68**	**E**	**K**	**1**	**2**	**1**	**2**	**1**	**3**	**n**	**n**	**1**	**2**	**1**	**1**	**1**	**2**	**2**	**2**	**2**	**3**	**3**	**3**
***holarctica*** ** FRAN012**	**e**	**K**	**1**	**2**	**1**	**2**	**1**	**3**	**n**	**n**	**1**	**2**	**1**	**1**	**1**	**2**	**2**	**2**	**2**	**3**	**3**	**3**
**KY99-3387**	**e**	**K**	**1**	**2**	**1**	**2**	**1**	**3**	**n**	**n**	**1**	**2**	**1**	**1**	**1**	**2**	**2**	**2**	**2**	**3**	**3**	**3**
*OSU18 [CP000437.1]*	e	E	1	2	1	5	1	3	Δ	Δ	1	2	1	1	1	2	2	2	2	3	3	3
*FSC200 [AASP00000]*	e	J	1	2	1	2	1	3	Δ	Δ	1	2	1	1	1	2	2	6	2	3	3	3
*FSC022 [AAYD00000]*	f	G	1	2	1	2	1	3	Δ	Δ	1	2	1	1	1	1	1	1	5	5	3	3
*URFT1 [ABAZ00000]*	g	H	1	2	1	2	1	3	Δ	Δ	1	2	2	4	1	2	1	1	3	6	3	3
*FTNF002-00 [CP00083.1]*	h	I	1	2	1	2	1	3	Δ	Δ	1	2	2	4	1	2	1	1	2	3	3	3
**425**	**n**	**M**	**1**	**2**	**1**	**4**	**1**	**3**	**n**	**n**	**1**	**2**	**1**	**1**	**1**	**2**	**4**	**4**	**2**	**3**	**4**	**4**
**OR96-03246**	**o**	**R**	**1**	**2**	**1**	**4**	**1**	**3**	**n**	**n**	**1**	**2**	**1**	**1**	**1**	**2**	**3**	**3**	**2**	**3**	**4**	**4**
**subspecies ** ***mediasiatica***			**FLT**	**GT**	**FLT**	**GT**	**FLT**	**GT**	**FLT**	**GT**	**FLT**	**GT**	**FLT**	**GT**	**FLT**	**GT**	**FLT**	**GT**	**FLT**	**GT**	**FLT**	**GT**
*FSC147 [CP000915.1]*	i	**N**	1	4	1	6	3	4	1	3	1	1	3	3	1	1	5	5	4	7	3	5
**subspecies ** ***novicida***			**FLT**	**GT**	**FLT**	**GT**	**FLT**	**GT**	**FLT**	**GT**	**FLT**	**GT**	**FLT**	**GT**	**FLT**	**GT**	**FLT**	**GT**	**FLT**	**GT**	**FLT**	**GT**
**KM14S**	**k**	**O**	**1**	**2**	**1**	**2**	**2**	**5**	**1**	**4**	**1**	**3**	**1**	**6**	**1**	**3**	**n**	**n**	**n**	**n**	**4**	**6**
**GB2**	**k**	**O**	**1**	**2**	**1**	**2**	**2**	**5**	**1**	**4**	**1**	**3**	**1**	**6**	**1**	**3**	**n**	**n**	**n**	**n**	**4**	**6**
**CG21**	**k**	**O**	**1**	**2**	**1**	**2**	**2**	**5**	**1**	**4**	**1**	**3**	**1**	**6**	**1**	**3**	**n**	**n**	**n**	**n**	**4**	**6**
*U112 [CP000439.1]*	k	O	1	2	1	2	2	5	1	4	1	3	1	6	1	3	Δ	Δ	Δ	Δ	4	6
*FTE [ABSS00000]*	k	O	1	2	1	2	2	5	1	4	1	3	1	6	1	3	Δ	Δ	Δ	Δ	4	6
*FTG [ABXZ00000]*	k	U	1	2	1	2	2	5	1	4	1	3	1	5	1	4	Δ	Δ	Δ	Δ	4	6
*Fx1 [CP002557]*	j	V	1	5	1	8	1	8	1	4	1	4	1	1	1	5	Δ	Δ	2	10	4	8
*3523 [CP002558]*	m	S	1	8	1	9	1	9	1	5	1	5	1	7	1	6	Δ	Δ	Δ	Δ	4	7
**GA99-3548 [ABAH00000]**	**l**	**P**	**1**	**6**	**1**	**8**	**1**	**7**	**1**	**6**	**1**	**4**	**1**	**5**	**1**	**4**	**3**	**7**	**2**	**8**	**n**	**n**
**GA99-3549 [AAYF00000]**	**p**	**Q**	**2**	**7**	**1**	**7**	**1**	**6**	**n**	**n**	**1**	**4**	**1**	**6**	**1**	**4**	**1**	**8**	**2**	**9**	**4**	**6**
***F. philomiragia***			**FLT**	**GT**	**FLT**	**GT**	**FLT**	**GT**	**FLT**	**GT**	**FLT**	**GT**	**FLT**	**GT**	**FLT**	**GT**	**FLT**	**GT**	**FLT**	**GT**	**FLT**	**GT**
**ATCC 25015 [ABYY00000]**	–	–	**n**	**n**	**n**	**n**	**n**	**n**	**1**	**8**	**n**	**n**	**n**	**n**	**n**	**n**	**n**	**n**	**n**	**n**	**n**	**n**
**ATCC 25016**	–	–	**n**	**n**	**n**	**n**	**n**	**n**	**1**	**9**	**n**	**n**	**n**	**n**	**n**	**n**	**n**	**n**	**n**	**n**	**n**	**n**
**ATCC 25017 [CP000937.1]**	–	–	**n**	**n**	**n**	**n**	**n**	**n**	**1**	**9**	**n**	**n**	**n**	**n**	**n**	**n**	**n**	**n**	**n**	**n**	**n**	**n**

Corresponding Genbank accession numbers are in brackets. FLT: Fragment Length Type; GT: sequence genotype of individual locus; Δ: strain is deficient of this locus; n: no PCR product or sequence detected.

Sequence analysis (including *in silico*) of fragments obtained from the 10 loci (Table 2) showed a total of 21 STs in the 41 *Ft* isolates (note, this number excludes the 3 *Fp* strains due to the lack of detectable sequence in 9 of 10 loci). No discrepancies between the focused sequencing data and published WGS data (23 strains available) were found. The five additional STs (as compared to 16 hAFLP types) based on sequence analysis are associated with subsp. *holarctica* strains and are due to SNPs and single basepair indels that are only detected by sequencing. Several of the sequenced DNAs originate from closely related derivatives or share a close geographical origin. As expected, STs are indistinguishable from parental strains in case of seven type A1 (subsp. *tularensis*) strains, which all have ST C. All of these strains were isolated in Illinois and may be from the same clonal origin. Interestingly, Scherm, another type A1 strain isolated in Ohio in 1944, also shares ST C.

The *Ft* 10-plex panel allows classification of strains by *Ft* subspecies: Type A1 strains have either ST A or C, and type A2 strains all share ST B. ST C is observed in all type A1 Illinois isolates, in strain Scherm (Ohio) and in NE061598 (Nebraska). Interestingly, strain Scherm and Schu S4, which are reportedly closely related [53] are distinguishable by their STs (ST C and ST A, respectively). Only three STs are observed in subsp. *tularensis,* but *subsp. holarctica* strains have much greater diversity with 11 STs. This is expected as these strains are isolated from the entire Northern hemisphere, as opposed to type A, which is, with some exceptions, concentrated in North America. Critically, there is no overlap in STs found in pathogenic *Ft* and the opportunistic *novicida* subspecies. Furthermore, the phylogenetically more distant species *Fp* readily distinguishable from *Ft* (both by fragment sizing and sequencing) as only one locus (i.e., *pdp*D) was amplifiable under the stringent assay conditions from *Fp* DNA. Figure S2 shows the phylogenetic tree based solely on the *Ft* 10-plex panel experimentally obtained gene sequences and whole genome sequence available data that have been concatenated after clustal alignment and visualization with PHYLIP (TreeView, [51]). This tree corroborates the clustering into *Ft* subspecies *tularensis, holarctica*, and *novicida* as explained above. Figure S3 shows a sample sequence trace from direct sequence analysis of *Ft fop*A using the 30-plex assay PCR amplicon.

### *Y. pestis* Multiplexed PCR Sizing and Rapid Focused Sequencing Assays

Published WGS data from 30 *Yp* and near neighbor strains were used to select 10 target loci for primer design. Targets were chosen to distinguish 1) *Yp* from *Ypst* and *Ye*; and 2) all *Yp* biovars. pCD is frequently detected in *Ypst* and *Ye* [54] and its presence is therefore not diagnostic of *Yp*. In addition, some *Ypst* strains also harbor other *Yp* or *Yp-*like plasmids or plasmid loci [55], stressing the importance of including both chromosomal and plasmid loci for discrimination of *Yp* from near neighbors *Ypst* and *Ye.* Six chromosomal and four plasmid-encoded targets (2 each encoded on pMT and pPCP) represent a mix of loci harboring SNPs, indels, and VNTR-like repeats. Primer sequences and target information are found in Table S3. The fully assembled 10-plex *Yp* panel was tested on 100 genome copy equivalents from each of 34 *Yp* strains representing the 3 biovars (14 *orientalis*, 13 *medievalis*, and 7 *antiqua*) and 10 near neighbor strains including *Ypst and Ye*. Five representative fragment sizing profiles of 4 *Yp* strains and one near neighbor *Ypst* strain are shown in Figure 3 (right panel).

Size variability in *Yp* 10-plex fragment panel is observed primarily in 4 loci (*glp*D, *ail, arg*S-YPO2047 and YPO1976). For example, *glp*D-specific amplicons are either 436 bp (*orientalis* biovar strain) or 529 bp (*antiqua* and *mediavalis* biovar strains) and are therefore useful in determining the presence or absence of biovar *orientalis* DNA, an important diagnostic marker for the only naturally occurring *Yp* biovar in North America. This is illustrated in Figure 3 (right panel), as strain Kuma, a bv. antiqua strain, shows the indicative long *glp*D amplicon as opposed to the short fragment in the bv. *orientalis* strains CO92, A1122 and Kimberley. The two VNTR-containing loci, *arg*S-YPO2047 and YPO1976, have the highest variability among all strains, 11 and 8 FLTs respectively, and contribute significantly to the overall genotypic resolution of the panel. The size-invariable plasmid encoded loci (*ymt, caf*1, and *pla*) and the variable *pst-*PCP06 assist in the characterization of *Yp* isolates that have lost plasmids or individual plasmid loci. Examples include the attenuated derivative of the virulent orientalis strain CO92, two avirulent derivatives of ori A1122, ori strain Dodson, a med KIM derivative (ATCC BAA-1612), and ant Pestoides F, all of which lack detectable pPCP-specific PCR products. Experimentally determined FLTs and hAFLP types from 34 *Yp* strains and 10 near neighbor species strains are listed in Table 3. The predicted FLTs and hAFLP types from an additional 17 *Yp* and 1 *Ypst* strain with published WGS data are also presented. A total of 33 hAFLP types were detected in the 51 *Yp* strains. The diversity is somewhat suppressed by the presence of derivatives of several parental strains than are each expected to have identical hAFLP types. These include strain KIM10 and 6 of its attenuated isolates all displaying hAFLP type o, and 2 derivatives each of strain Kimberley (hAFLP type g), Kuma (hAFLP type y) and A12 (hAFLP type c). The chromosomal loci selected for the 10-plex *Yp* panel are predicted to be present in *Ypst* species and amplifiable by the primers designed based on available WGS data. This was confirmed experimentally, and all six chromosomal loci amplified in *Ypst*, most with FLTs indistinguishable from those observed in the *Yp* species. Exceptions are the VNTR-harboring loci *arg*S-YPO2407 and YPO1976, which have FLTs not observed in *Yp* (5 of 5 *Ypst arg*S-YPO2047 and 3 of 5 *Ypst* YPO1976 FLTs, respectively). Furthermore, sequences of these chromosomal loci readily distinguish *Ypst* from *Yp* strains (Table 3). Critically, none of the *Ypst* strain DNAs yielded pMT and pPCP amplicons and could readily be distinguished from *Yp* strains. In contrast, the 10 *Yp* loci are either absent from or cannot be amplified by the *Yp* primers in the other near neighbor species including *Ye, Y. aldovae, Y. kristensenii, Y. frederiksenii, Y. ruckeri, and Y. rohdei*. As expected, none of the *Yp* 10-plex loci amplified from the three analyzed *Ye* strains nor from *Y. aldovae, Y. frederiksenii* and *Y. kristensenii*.

**Table 3:** hAFLP types and Sequence Types (STs) of *Yp* strains and *Yp* near neighbor species DNA (shown in bold) and by *in silico* analysis of WGS strains (shown in italics).

Strain [GenbankAcc#]	hAFLP	ST	*glg*X		*glp*D		*ail*		*nuo*G		*arg*S-2407		YPO1976		MT_*ymt*		MT_*caf*1		PCP_*pla*		PCP_*pst*	
*biovar orientalis:*			FLT	GT	FLT	GT	FLT	GT	FLT	GT	FLT	GT	FLT	GT	FLT	GT	FLT	GT	FLT	GT	FLT	GT
**CO92 [AL590842.1]**	**a**	**A**	**1**	**1**	**1**	**1**	**1**	**1**	**1**	**1**	**1**	**1**	**1**	**1**	**1**	**1**	**1**	**1**	**1**	**1**	**1**	**1**
**CO92, attenuated**	**d**	**D**	**1**	**1**	**1**	**1**	**1**	**1**	**1**	**1**	**1**	**1**	**1**	**1**	**1**	**1**	**1**	**1**	**n**	**n**	**n**	**n**
*CA88-4125 [ABCD000000]*	a	A	1	1	1	1	1	1	1	1	1	1	1	1	1	1	1	1	1	1	1	1
**A1122**	**b**	**B**	**1**	**1**	**1**	**1**	**1**	**1**	**1**	**1**	**5**	**5**	**1**	**1**	**1**	**1**	**1**	**1**	**1**	**1**	**1**	**1**
**A12**	**c**	**C**	**1**	**1**	**1**	**1**	**1**	**1**	**1**	**1**	**5**	**5**	**1**	**1**	**1**	**1**	**1**	**1**	**n**	**n**	**n**	**n**
**A12**	**c**	**C**	**1**	**1**	**1**	**1**	**1**	**1**	**1**	**1**	**5**	**5**	**1**	**1**	**1**	**1**	**1**	**1**	**n**	**n**	**n**	**n**
**Java 9**	**e**	**E**	**1**	**1**	**1**	**1**	**1**	**1**	**1**	**1**	**10**	**10**	**1**	**1**	**n**	**n**	**n**	**n**	**1**	**1**	**1**	**1**
**PBM19**	**f**	**F**	**1**	**1**	**1**	**1**	**1**	**1**	**1**	**1**	**10**	**10**	**1**	**1**	**1**	**1**	**1**	**1**	**1**	**1**	**1**	**1**
**Shasta**	**g**	**G**	**1**	**1**	**1**	**1**	**1**	**1**	**1**	**1**	**6**	**6**	**1**	**1**	**1**	**1**	**1**	**1**	**1**	**1**	**1**	**1**
**Dodson**	**h**	**H**	**1**	**1**	**1**	**1**	**1**	**1**	**1**	**1**	**4**	**4**	**1**	**1**	**1**	**1**	**1**	**1**	**n**	**n**	**n**	**n**
**El Dorado**	**i**	**I**	**1**	**1**	**1**	**1**	**1**	**1**	**1**	**1**	**3**	**3**	**3**	**3**	**1**	**1**	**1**	**1**	**1**	**3**	**1**	**1**
**Kimberley**	**g**	**G**	**1**	**1**	**1**	**1**	**1**	**1**	**1**	**1**	**6**	**6**	**1**	**1**	**1**	**1**	**1**	**1**	**1**	**1**	**1**	**1**
**Kimberley**	**g**	**G**	**1**	**1**	**1**	**1**	**1**	**1**	**1**	**1**	**6**	**6**	**1**	**1**	**1**	**1**	**1**	**1**	**1**	**1**	**1**	**1**
**TS**	**o**	**O**	**1**	**1**	**2**	**2**	**1**	**1**	**1**	**1**	**4**	**4**	**2**	**2**	**1**	**1**	**1**	**1**	**1**	**1**	**1**	**2**
**TS**	**b**	**B**	**1**	**1**	**1**	**1**	**1**	**1**	**1**	**1**	**5**	**5**	**1**	**1**	**1**	**1**	**1**	**1**	**1**	**1**	**1**	**1**
*India 195 [ACNR000000]*	k	K	1	1	1	1	1	1	1	1	4	4	1	1	1	1	1	1	1	1	1	1
*IP275 [AAOS000000]*	k*	K*	1	1	1	1	#	#	1	1	4	4	1	1	1	1	1	1	1	1	1	1
*F1991016 [ABAT000000]*	l	L	1	1	1	1	1	1	1	1	3	3	1	1	1	1	1	1	1	1	1	1
*MG05-1010 [AAYS000000]*	m	M	1	1	1	1	1	1	1	1	1	1	2	2	1	1	1	1	1	1	1	1
*PEXU2 [ACNS000000]*	n	N	1	1	1	1	1	1	1	1	1	1	4	4	1	1	1	1	1	1	1	1
*FV-1 [AAUB000000]*	l	FF	1	1	1	1	1	7	1	2	3	3	1	1	1	1	1	1	1	1	1	1

*biovar mediaevalis:*			FLT	GT	FLT	GT	FLT	GT	FLT	GT	FLT	GT	FLT	GT	FLT	GT	FLT	GT	FLT	GT	FLT	GT
**KIM 10v [AE009952]**	**o**	**O**	**1**	**1**	**2**	**2**	**1**	**1**	**1**	**1**	**4**	**4**	**2**	**2**	**1**	**1**	**1**	**1**	**1**	**1**	**1**	**2**
**KIM D27 [ADDC000000]**	**p**	**P**	**1**	**1**	**2**	**2**	**1**	**1**	**1**	**3**	**4**	**4**	**2**	**2**	**2**	**2**	**1**	**1**	**1**	**1**	**2**	**3**
**KIM 5**	**o**	**O**	**1**	**1**	**2**	**2**	**1**	**1**	**1**	**1**	**4**	**4**	**2**	**2**	**1**	**1**	**1**	**1**	**1**	**1**	**1**	**2**
**Kim, attenuated**	**b**	**B**	**1**	**1**	**1**	**1**	**1**	**1**	**1**	**1**	**5**	**5**	**1**	**1**	**1**	**1**	**1**	**1**	**1**	**1**	**1**	**1**
**Kim, attenuated**	**o**	**O**	**1**	**1**	**2**	**2**	**1**	**1**	**1**	**1**	**4**	**4**	**2**	**2**	**1**	**1**	**1**	**1**	**1**	**1**	**1**	**2**
**Kim, attenuated**	**q**	**Q**	**1**	**1**	**2**	**2**	**1**	**1**	**1**	**1**	**4**	**4**	**2**	**2**	**1**	**1**	**1**	**1**	**n**	**n**	**n**	**n**
**Kim, attenuated**	**o**	**O**	**1**	**1**	**2**	**2**	**1**	**1**	**1**	**1**	**4**	**4**	**2**	**2**	**1**	**1**	**1**	**1**	**1**	**1**	**1**	**2**
**K25 D21**	**o**	**O**	**1**	**1**	**2**	**2**	**1**	**1**	**1**	**1**	**4**	**4**	**2**	**2**	**1**	**1**	**1**	**1**	**1**	**1**	**1**	**2**
**K25**	**o**	**O**	**1**	**1**	**2**	**2**	**1**	**1**	**1**	**1**	**4**	**4**	**2**	**2**	**1**	**1**	**1**	**1**	**1**	**1**	**1**	**2**
**K25**	**o**	**O**	**1**	**1**	**2**	**2**	**1**	**1**	**1**	**1**	**4**	**4**	**2**	**2**	**1**	**1**	**1**	**1**	**1**	**1**	**1**	**2**
*Pestoides A* [ACNT000000]	r	R	1	1	2	2	1	3	1	1	5	5	4	4	1	1	1	1	1	2	4	4
**Pestoides B**	**r**	**R**	**1**	**1**	**2**	**2**	**1**	**3**	**1**	**1**	**5**	**5**	**4**	**4**	**1**	**1**	**1**	**1**	**1**	**2**	**4**	**4**
**Harbin 35 [CP001608]**	**s**	**S**	**1**	**1**	**2**	**2**	**1**	**6**	**1**	**1**	**10**	**10**	**8**	**8**	**1**	**1**	**1**	**1**	**1**	**1**	**1**	**2**
**Nicholisk 41**	**s**	**S**	**1**	**1**	**2**	**2**	**1**	**6**	**1**	**1**	**10**	**10**	**8**	**8**	**1**	**1**	**1**	**1**	**1**	**1**	**1**	**2**
*K1973002 [AAYT000000]*	t*	T*	1	1	2	2	1	1	1	1	4	4	2	2	1	1	#	#	#	#	#	#

*biovar antiqua:*			FLT	GT	FLT	GT	FLT	GT	FLT	GT	FLT	GT	FLT	GT	FLT	GT	FLT	GT	FLT	GT	FLT	GT
**Antiqua [CP000308.1]**	**u**	**U**	**1**	**1**	**2**	**2**	**1**	**1**	**1**	**1**	**6**	**6**	**1**	**1**	**1**	**1**	**1**	**1**	**1**	**1**	**1**	**1**
**Pestoides F [CP000668.1]**	**v**	**V**	**1**	**1**	**2**	**2**	**2**	**2**	**1**	**1**	**9**	**9**	**6**	**6**	**1**	**1**	**1**	**2**	**n**	**n**	**n**	**n**
**Angola [CP000901.1]**	**w**	**W**	**1**	**2**	**2**	**2**	**1**	**5**	**1**	**1**	**11**	**11**	**1**	**1**	**1**	**1**	**n**	**n**	**1**	**2**	**2**	**7**
**Nairobi**	**x**	**X**	**1**	**3**	**2**	**2**	**1**	**1**	**1**	**1**	**2**	**2**	**5**	**5**	**n**	**n**	**n**	**n**	**1**	**1**	**1**	**1**
**Yokohama**	**y**	**Y**	**1**	**1**	**2**	**2**	**1**	**6**	**1**	**1**	**10**	**10**	**8**	**8**	**1**	**1**	**1**	**1**	**1**	**1**	**3**	**6**
**Kuma**	**y**	**Y**	**1**	**1**	**2**	**2**	**1**	**6**	**1**	**1**	**10**	**10**	**8**	**8**	**1**	**1**	**1**	**1**	**1**	**1**	**3**	**6**
**Kuma**	**y**	**Y**	**1**	**1**	**2**	**2**	**1**	**6**	**1**	**1**	**10**	**10**	**8**	**8**	**1**	**1**	**1**	**1**	**1**	**1**	**3**	**6**
*B42003004 [AAYU000000*	z	**Z**	1	1	2	2	1	1	1	1	4	4	4	4	1	1	1	1	1	1	1	1
*UG05-0454 [AAYR000000]*	aa	**AA**	1	1	2	2	1	1	1	1	7	7	1	1	1	1	1	1	1	1	1	1
*E1979001 [AAYV000000]*	bb	**BB**	1	1	2	2	1	1	1	1	8	8	5	5	1	1	1	1	1	1	1	1
*D182038 [CP01589]*	gg	**GG**	1	1	2	2	1	1	1	1	7	7	5	5	1	1	1	1	1	1	1	1
*Z176003 [CP001593.1]*	cc	**CC**	1	1	2	2	1	1	1	1	4	4	1	1	1	1	1	1	1	1	4	5
*D106004 [CP001585]*	dd	**DD**	1	1	2	2	1	1	1	1	9	9	7	7	1	1	1	1	1	1	1	1
*Nepal 516 [CP000305.1]*	j	**J**	1	1	2	2	1	6	1	1	3	3	8	8	1	1	1	1	1	1	3	6

*biovar microtus:*			FLT	GT	FLT	GT	FLT	GT	FLT	GT	FLT	GT	FLT	GT	FLT	GT	FLT	GT	FLT	GT	FLT	GT
91001	ee	EE	1	1	2	2	1	4	1	1	1F	12	1	1	1	1	1	1	1	2	4	4

Near Neighbor strains:			FLT	GT	FLT	GT	FLT	GT	FLT	GT	FLT	GT	FLT	GT	FLT	GT	FLT	GT	FLT	GT	FLT	GT
***Ypst*** ** YPIII [CP000950.1]**	**Na**	**NA**	**1**	**1**	**2**	**3**	**1**	**8**	**1**	**6**	**13**	**13**	**2**	**11**	**n**	**n**	**n**	**n**	**n**	**n**	**n**	**n**
***Ypst*** ** PB1/+ [CP001048.1]**	**Nb**	**NB**	**1**	**4**	**2**	**3**	**1**	**9**	**1**	**4**	**14**	**14**	**1**	**1**	**n**	**n**	**n**	**n**	**n**	**n**	**n**	**n**
*Ypst IP31758* [CP000720.1]	Nc	NC	1	4	2	3	1	8	1	5	15	15	10	14	Δ	Δ	Δ	Δ	Δ	Δ	Δ	Δ
***Ypst*** ** IP32953 [BX396398.1 ]**	**Nd**	**ND**	**1**	**4**	**2**	**3**	**1**	**8**	**1**	**4**	**14**	**14**	**9**	**9**	**n**	**n**	**n**	**n**	**n**	**n**	**n**	**n**
***Ypst*** ** Pa3606**	**Ne**	**NE**	**1**	**4**	**2**	**3**	**1**	**8**	**1**	**7**	**11**	**16**	**10**	**10**	**n**	**n**	**n**	**n**	**n**	**n**	**n**	**n**
***Ye*** ** Billups 1803-68**	–	–	**n**	**n**	**n**	**n**	**n**	**n**	**n**	**n**	**n**	**n**	**n**	**n**	**n**	**n**	**n**	**n**	**n**	**n**	**n**	**n**
***Ye*** ** WA**	–	–	**n**	**n**	**n**	**n**	**n**	**n**	**n**	**n**	**n**	**n**	**n**	**n**	**n**	**n**	**n**	**n**	**n**	**n**	**n**	**n**
***Ye*** ** 8081 [AM286415.1]**	–	–	**n**	**n**	**n**	**n**	**n**	**n**	**n**	**n**	**n**	**n**	**n**	**n**	**n**	**n**	**n**	**n**	**n**	**n**	**n**	**n**
***Y. aldovae*** ** 670-83**	–	–	**n**	**n**	**n**	**n**	**n**	**n**	**n**	**n**	**n**	**n**	**n**	**n**	**n**	**n**	**n**	**n**	**n**	**n**	**n**	**n**
***Y. frederiksenii*** ** Y225**	–	–	**n**	**n**	**n**	**n**	**n**	**n**	**n**	**n**	**n**	**n**	**n**	**n**	**n**	**n**	**n**	**n**	**n**	**n**	**n**	**n**
***Y. kristensenii*** ** Y231**	–	–	**n**	**n**	**n**	**n**	**n**	**n**	**n**	**n**	**n**	**n**	**n**	**n**	**n**	**n**	**n**	**n**	**n**	**n**	**n**	**n**

Corresponding Genbank accession numbers are in brackets. FLT: Fragment Length Type; GT: sequence genotype of individual locus; Δ: strain is deficient of this locus; n: no PCR product and no sequence detected; t: strain FV-1 has 3 copies of pPCP-*pla* locus in whole genome data belonging to GT 1 and 4;

* hAFLP Type and ST assigned despite missing data (marked with “#”) in the WGS data.

Sequence analysis of fragments obtained from all 10 *Yp* panel loci showed a total of 34 STs in the 51 isolates (Table 3). The additional ST (as compared to hAFLP types) distinguishes strain ori FV-1 (ST FF) from ori F1991016 (ST L). The nearly identical number of hALFP types and STs is due primarily to the convergence of genotypes detected in the VNTR-repeat containing loci *arg*S-YPO2047 and YPO1976 (12 and 8, respectively) as no polymorphisms beyond the VNTR repeat number variation are present in the amplified fragments. In contrast, 7 locus GTs compared to only 4 FLTs are present in pPCP-encoded *pst*-PCP06 because sequencing detects synonymous insertions and deletions that do not affect fragment size. Previously unreported GTs (and consequently STs) due to SNPs were detected for the *glg*X locus in strain Nairobi (GT 3) and the pPCP *pla* locus of strain ori El Dorado (GT 3). WGS data from 7 of the 34 *Yp* strains are published, and no discrepancies between *in silico* predicted and fragment size genotypes derived by 10-plex PCR were discovered.

The variability observed in both the multiplexed PCR sizing and Rapid Focused Sequencing assays results in almost complete resolution of all analyzed *Yp* strains. Strains that are not resolvable by ST are 7 closely-related strain KIM derivatives (ST O), 2 derivatives of strain Kuma (ST Y), and 2 of strain Kimberley (ST G). Among unrelated isolates, only the enzooic central Asian isolates Pestoides A and B (both ST R), the Chinese strains Harbin 35 and Nicholisk 41 (both ST S), the attenuated Kimberley strains (South African origin) and North American strain Shasta (ST G), and the attenuated derivatives of strains Yokohama and Kuma (ST Y) are not distinguishable by Rapid Focused Sequencing using the current 10-plex PCR *Yp* panel fragments. Sequence analysis demonstrated that the close near neighbors *Ypst* YPIII, PB1/+, Pa3606, IP32953, and IP31758 are unambiguously distinguishable from *Yp* by: a) the absence of pMT and pPCP plasmid-specific loci; b) distinct STs that are not found in any of the *Yp* strains. Finally, two derivative strains belonging to different biovars, bv. *mediaevalis* KIM BAA1504 (Kim, attenuated with hAFLP type b and ST B) and bv. *orientalis* TS BAA1505 (TS with hAFLP type o and ST O) have opposite identities as shown in Table 3. These two strains were very likely switched at the source or following deposit to ATCC. Figure S4 shows the phylogenetic tree based solely on the *Yp* 10-plex panel experimentally obtained gene sequences and whole genome sequence available data that have been concatenated after clustal alignment and visualization with PHYLIP (TreeView, [51]).

Specificity of the 10-plex panel was evaluated by testing the assay against 10^5^ copies of closely-related species *Y. pseudotuberculosis, Y. kristensenii, Y. aldovae,* and *Y. enterocolitica.* In addition, assays were performed with the four near-neighbors present in 100-fold molar excess of the target pathogens (Figures S5A–5D). In the presence of 10^5^ genome equivalents of *Ypst* YPIII DNA, all 6 chromosomal loci are amplified (Figure S5A, left panel) as expected. However, the length of the *Ypst arg*S amplicon is distinguishable from that of *Yp* strains and the 4 *Yp* plasmid targets are not amplified, clearly indicating that *Yp* is not present. When *Ypst* DNA is spiked in 100-fold excess to KIM10 DNA, the chromosomal targets of this strain are out-competing the targets of KIM10. However, all expected *Yp* KIM10 loci are still present, albeit at a low level (Figure S5A, right panel). In the presence of 10^5^ genome equivalents of *Yk* CDC1457-B1, no loci are amplified (Figure S5B, left panel), and a 100-fold molar excess of *Yk* DNA relative to *Yp* KIM10 DNA does not alter the KIM10 profile (Figure S5B, right panel). For *Yal* 670-B1, 10^5^ genome equivalents generate only a low-intensity peak for *nuo*G (Figure S5C, left panel). When *Yal* DNA is spiked in 100-fold molar excess to KIM10 DNA, all specific *Yp* loci are still clearly identifiable (Figure S5C, right panel). Finally, at 10^5^ genome equivalents of *Ye* WA DNA, no *Yp* specific products are observed in the profile (Figure S5D, left panel), and the addition of 100-fold molar excess of *Ye* DNA also does not disturb the KIM10 profile (Figure S5D, right panel). Taken together, these data suggest that specific target organism can be unambiguously identified even in the presence of closely-related species in molar excess.

### *Ba-Ft-Yp* 30-plex

**Figure 4:**
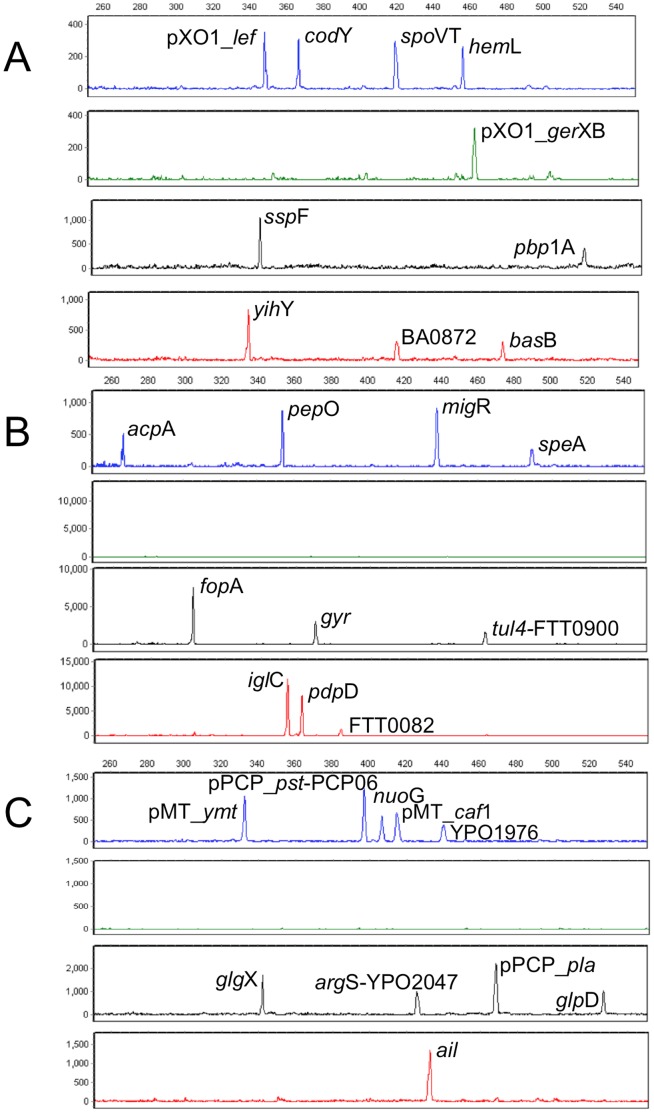
30-plex PCR profiles generated from select biothreat agents *Ba, Ft*, and *Yp*. Electropherogram obtained from 100 copies of *Ba* strain Sterne (A); from 1000 copies of *Ft* subsp. *tularensis* WY96 (B); and from 100 copies of *Yp bv. mediavalis* strain KIM10v (C). Note that 1000 copies of *Ft* DNA are required to detect two of the FAM-labeled targets (*acp*A and *spe*A) due to their high AT content resulting in reduced PCR efficiency.

We have combined 60 primers (20 from each of the *Ba*, *Ft*, and *Yp* 10-plex PCR assays) and performed an initial evaluation of the resulting 30-plex panel in the presence of 100 genome copy equivalents from *Ba* and *Yp* and 1000 from *Ft* representatives of each species. The resulting electropherograms using *Ba* Sterne, *Yp* med KIM10v, and *Ft* subsp. *tularensis* Schu S4 templates are shown in Figure 4. The 30-plex fragment sizing profiles are identical with the individual 10-plex assays (e.g. the profiles of Sterne in Figure 2, WY96 and KIM10v in Figure 3). The electropherograms for each species are readily distinguished, with each biothreat species displaying a characteristic signature. In addition, certain fragments such as a full length (526 bp) *Yp glp*D amplicon (KIM10v) or a 361 bp *Ft pdp*D amplicon (SchuS4), correctly indicate the presence of *Yp* bv. *mediaevalis* or *antiqua* strain and a subsp. *tularensis* strain, respectively. For *Ft*, 1000 copies of template DNA are required to detect two of the FAM-labeled targets (*acp*A and *spe*A). This is due to the fact that *Ft* DNA has the lowest GC content (32%) of all three species (*Ba* 35% and *Yp* 48%). Primers for these two loci have high AT content which resulted in reduced PCR efficiency due to slower annealing kinetics. Primer redesign for these two *Ft* loci is in progress.

The specificity of 30-plex panel was evaluated against genomic DNAs from 14 environmental background species (EBS, 10^3^ genome equivalents per strain): *Clostridium perfringens* ATCC 13124, *Staphylococcus aureus* ATCC 10832, *Pseudomonas aeruginosa* Boston 41501, *Burkholderia cepacia* 249, *Fusobacterium nucleatum* 1612A, *Bacteroides fragilis* NCTC 9343, *Streptococcus pneumoniae* TIGR4, *Shewanella oneidensis* MR-1, *Synechocystis* sp. PCC 6308, *Lactobacillus plantarum* 17-5, *Listeria monocytogenes* EGDe, *Bacillus megaterium* ATCC 14581, *Chryseobacterium indologenes* NCTC 10796, and *Bacillus psychrosaccharolyticus* T25B. No detectable PCR products have been observed in the fragment sizing profiles from any of these species. Figure S6A is a representative profile detecting only background noise from all EBS. To further evaluate specificity and sensitivity of the 30-plex assay, target DNAs were spiked with 10^6^ copies of EBS. In the presence of this high copy number EBS, the 10-plex signatures of the biothreat agents remained unaffected. Figure S6 (B and C) shows the simultaneous amplification of the 10 *Ba* loci (100 copies of Sterne) and 10 *Yp* loci (100 copies of Kim10) from the 30-plex panel in the presence of EBS (10^6^ copies of *B. cepacia* 249). Taken together, these data demonstrate specificity of the designed primers and sensitivity of the 30-plex panel in a large molar excess of background DNA. This data confirms and extends the power of the multiplexed assay to identify and strain type biothreats while clearly discriminating against closely-related species and environmental background strains.

**Figure 5:**
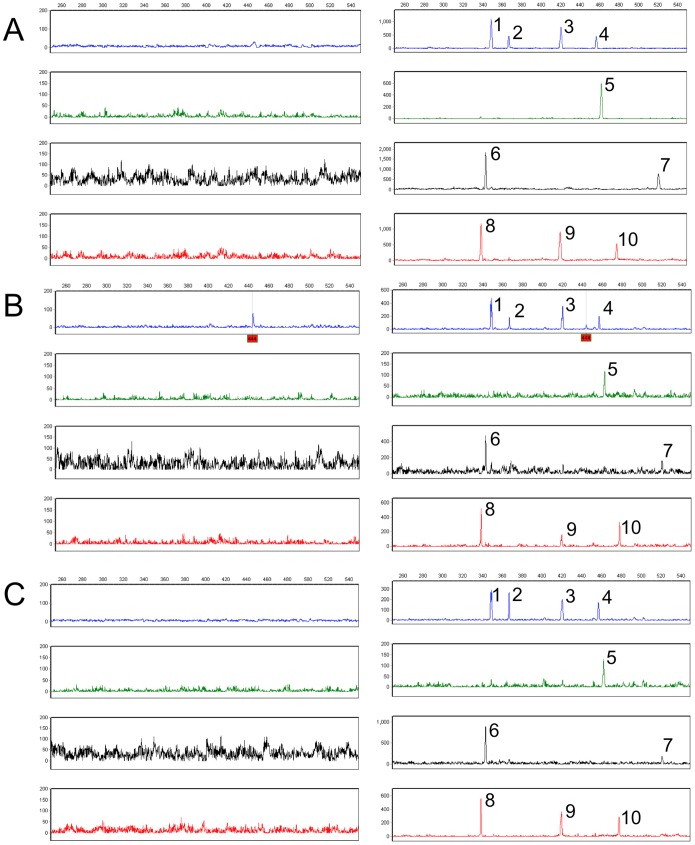
Efficiency and sensitivity of the 30-plex panel with real-world air filter samples. Electropherograms obtained from Pentagon filter (A); BioWatch filter I (B); and BioWatch filter II (C). Resulting profiles on the left were from amplification of DNAs purified from air filter; profiles on the right were from air filter DNAs spiked with 100 copies of *Ba* Sterne. Background noise was detected from direct amplification of the three air filter samples, and the 10-plex *Ba*-signature was generated for all *Ba*-spiked air filter DNA samples. The observed nonspecific peak at 444 bp (highlighted in red) was readily distinguished from the 10 specific product peaks (1-pXO1_*lef,* 2-*cod*Y, 3-*spo*VT, 4-*hem*L, 5-pXO1_*ger*XB, 6-*ssp*F, 7-*pbp*1A, 8-*yih*Y, 9-BA0872, and 10-*bas*B).

Finally, purified DNA was obtained from two types of real-world air filters (from the Pentagon Protection Agency and BioWatch, both acquired with the assistance of DHS S&T). These DNA samples were characterized for the presence of inhibitors based on 16S rDNA gene amplification with universal bacterial primers. Percent PCR inhibition was assessed from the intensities of the 16S PCR band on ethidium bromide-stained agarose gel resulting from amplification of air filter DNAs spiked with and without a known concentration of *B. subtilis* DNA as templates. Purified DNAs from one Pentagon and from two BioWatch (I, II) air filters were found to have PCR inhibition of 0%, 6% and 21%, respectively. The 30-plex panel was then tested using DNA purified from the three air filters and with air filter DNAs spiked with 100 copies of *Ba* Sterne. Resulting electropherograms are shown in Figure 5. Background noise was detected for all air filter samples without spiked *Ba* and the 10-plex *Ba*-signature was observed for all *Ba*-spiked air filter DNA samples. A very low nonspecific 444 bp peak was also observed in one BioWatch sample which was readily distinguished from the 10 specific product peaks. This initial result suggests that the purification, amplification, and electrophoresis protocols described here can be used to test DNA purified from environmental samples.

## Discussion

We have developed two related assays, a multiplexed PCR sizing assay and its derivative, a Rapid Focused Sequencing assay. Both assays enable the detection, identification, and characterization of biothreat agents, demonstrated here by the simultaneous interrogation of 30 loci, 10 each from *Ba, Ft*, and *Yp*. The primary difference between the two assays is that Rapid Focused Sequencing reveals additional variations (SNPs, size neutral indels) that do not lead to fragment size alterations. Sequence information of continuous 500 base fragments from 10 chromosomally and plasmid-encoded loci is in effect a self-verifying assay, providing a level of confidence that is not achievable by quantitative-PCR in conjunction with probe- or melt-curve-based or microarray-based detection. In addition, focused sequence analysis can detect novel mutations/polymorphisms that are indispensable for identification of novel strains. By sequencing approximately ten carefully selected loci per genome, the approach allows the precision of whole genome sequencing but is much simpler and amenable to rapid and autonomous detection. Furthermore, the accuracy of Sanger sequencing is far better than whole genome sequencing methods [56]–[58]. Taken together, we believe that the advantages of Rapid Focused Sequencing render the approach well-suited to biothreat detection in the field.

Rapid Focused Sequencing allows identification and strain differentiation of the biothreat agents and clear discrimination from closely-related species and environmental background strains. It will be critical to expand the number of biothreats detected to approximately two-dozen species. This expansion will require two parallel assays, an estimated 120 targets for a total of 12 DNA-based species and a similar number for RNA-based organisms. Within the microfluidic biochip, purified nucleic acid will therefore be divided into one multiplexed PCR and one multiplexed reverse transcription-PCR reaction prior to sequencing. This approach is likely feasible based on our previous work developing microfluidic RT-PCR and multiplexed assays based on 84 primer pairs (data not shown). However, it is noted that as the number of targets grows, artifacts generated by unrelated primer pairs must be designed out of the assay.

Each of the 3 10-plex PCR biothreat agent panels was validated by analyzing genomic DNA isolated from 20 *Ba,* 30 *Ft* and 34 *Yp* strains as well as DNA from 10 representatives each of their respective near neighbors strains or closely-related species. The choice of loci and the primer design warrants correct discrimination of non-pathogenic near neighbors from biothreat strains and allows identification of species and characterization into subtypes, biovars and potentially attenuated strains. In contrast to MLST methods, the Rapid Focused Sequencing approach is not limited to selectively neutral housekeeping genes but instead also utilizes virulence factor, VNTR-like, non-coding, and other targets. The development of an appropriate set of targets allows powerful identification and genotyping of pathogens of interest.

The total sequence information analyzed via the 10-plex PCR *Ba* panel represents approximately 0.07% of the ∼5.8 Mbp *Ba* genome (including chromosome and plasmids), 0.19% of the ∼2 Mbp *Ft* genome, and 0.09% of the ∼4.6 Mbp *Yp* genome. Rapid Focused Sequencing of this small fraction of the *Ba* genome, for example, is sufficient to determine presence or absence of the pathogen itself, to distinguish and characterize the majority of *Ba* strains by virulence (presence/absence of plasmid loci), and to unambiguously distinguish *Ba* strains from near neighbors. Similarly, Rapid Focused Sequencing of the *Ft* genome is sufficient to determine presence or absence of *Ft*-specific DNA, to distinguish between subspecies (*tularensis* and *holarctica*), and between subsp. *tularensis* type A1 and A2 strains. The approach also unambiguously distinguishes the near neighbors, subsp. *novicida,* from from pathogenic *Ft* subspp. *tularensis* and *holarctica* strains. The number and nature of the biothreat targets can be modified as the understanding of the genetics and biology of the pathogens advances over time.

The ability to perform the highly multiplexed amplification and Sanger sequencing reactions in microfluidic biochips offers the potential to perform these assays in the field, on both environmental and clinical samples. The choice of assay is dependent on the given concept of operation. For example, in certain clinical settings, fewer near neighbors may be present, and the multiplexed PCR assay may provide sufficient diagnostic information. In most environmental settings, however, the potential for an enormous variety of near neighbors and the need for forensic analysis may lead to the preferential use of the sequencing assay. We have recently developed a ruggedized system that incorporates nucleic acid purification, multiplexed amplification, and electrophoretic separation in a single microfluidic biochip. We believe that this foundational system can be adapted to perform both the multiplexed PCR sizing and Rapid Focused Sequencing assays. A fully integrated microfluidic platform that enables rapid pathogen analysis in the field has the potential to dramatically improve biothreat detection capabilities.

## Materials and Methods

### DNA Isolates and Dilutions

Isolated DNAs from biothreat strains, near neighbor strains and environmental background strains was obtained from ATCC (Manassas, VA), BEI Resources (Manassas, VA) or CRP (Critical Reagents Program, APG-EA, MD). Specifically, DNA from *Ba* strains Ames, Ames 35, PAK-1, Vollum 1B, BA1035, RA3, Sterne (NR-10310), Sterne (NR-10311), Weybridge (NR-10446), *Bt* strains Al Hakam, *konkukian* 97-27, *Bc* strains E33L, 3A, G9241, *B. megaterium* (DD-421), *Ft* subsp. *tularensis, holarctica, novicida* strains SchuS4, FRAN001, MA00-2987, LVS, 425, WY96, LVSG, 14LVS, KY99-3387, OR96-03246, KM14S, GB2, CG21, *F. philomiragia* ATCC2516, *Yp* strains CO92, KIM10v, Antiqua, Pestoides F, Java 9, A1122, *Y. pseudotuberculosis* YPIII, and *Y. entercocolitica* WA was from BEI Resources. DNAs from *B. coagulans* ATCC7050, *F. philomiragia* ATCC25017, attenuated *Yp* strains A12 (BAA-1506), A12 (BAA-1613), Kimberley (BAA-1509), Kimberley (BAA-1691), TS (BAA-1505), TS (BAA-1612), Kuma (BAA-1507), Kuma (BAA-1614), Yokohama (BAA-1508), K25 D21 (BAA-1511), K25 (BAA-1621), K25 (BAA-1622), KIM (BAA-1504), KIM (BAA-1596), KIM (BAA-1608), KIM (BAA-1611), *Y. aldovae* 670-83, and *Y. enterocolitica* Billups 1803-68 were obtained from ATCC. DNAs from *Ba* strains K1219 (Canadian Bison), V770-NP-1R, SK-102, 2002013094, Pasteur, Sterne (K7816), Turkey #32, Zimbabwe 89, K1811, A0370, *Bt* strains *israelensis* 35646, *kurstaki* HD1, *Bc* strain 4342, *Ft* subsp. *tularensis, holarctica, novicida* strains Scherm, FRAN005, FRAN006, FRAN007, FRAN008, FRAN009, FRAN011, FRAN015, VT68, FRAN012, JAP, D9876 (GA99-3548), F6168 (GA99-3549), *F. philomiragia* ATCC25015, *Yp* strains Pestoides B, Angola, Nairobi, Harbin 35, PBM19, Nicholisk 41, Shasta, Dodson, El Dorado, *Y. pseudotuberculosis* strains PB1/+, IP32953, PA3606, *Y. enterocolitica* 8081, *Y. frederiksenii* Y225, and *Y. kristensenii* Y231 was from CRP. DNA from *Ba* strain Sterne_2 was generously provided to NetBio by Dr. Timothy Read. DNA concentrations and purity were assessed using a NanoDrop spectrophotometer (Thermo Fisher, Waltham, MA). DNA stock concentrations (defined as the average of triplicate measurements) were diluted to 1 ng/µL in TE-4 buffer, pH 8 (10 mM Tris-HCl, 0.1 mM EDTA). Copy number was calculated using genome size of the sequenced reference strain and verified by testing serial dilutions in singleplex PCR.

### Primer Design

*Ba* Ames, *Ft* SchuS4 and *Yp* CO92 were selected as reference strains and loci of interest as published in the finished genomes were selected to search for homologous sequences in Genbank databases nr and WGS using blast [59]. Homologous sequences were aligned via ClustalX 2.1 [60] and conserved regions were identified for primer design. Primers were designed and tested *in silico* using VisualOMP (DNA Software, Ann Arbor, MI) and evaluated for specificity *in silico* via blast against Genbank nr and WGS, and by Thermoblast (DNA Software, Ann Arbor, MI) against selected near neighbor genome sequences.

### Primer Evaluation by Microfluidic Singleplex PCR, Assembly of the Microfluidic Multiplex Panel, and Microfluidic Electrophoresis

Individual primer pairs designed for each target loci were initially tested for performance by microfluidic singleplex PCR using 100 genome equivalents of reference strain template DNAs representing each agent and using only half of the total PCR reaction for separation and detection. The equivalent 50 copies for detection with high sensitivity as judged by PCR peak intensity allow better screening of primers for use in the multiplex panel assembly. The pXO2-deficient Sterne strain Sterne_2, med KIM derivative (ATCC BAA-1504), and subsp. *tularensis* WY96, were used as reference DNA strains for *Ba*, *Yp*, and *Ft*, respectively. The microfluidic biochip and rapid thermal cycler have been utilized for forensic [13], clinical diagnostics (manuscript for submission), and biothreat [61] applications. Seven microliters of reaction mixtures were amplified in 20 minutes using a 33-cycle protocol. Following amplification, PCR products were retrieved and 2.7 µL aliquots were microfluidically separated and detected by laser-induced fluorescence using the Genebench-FX instrument and accompanying biochip as previously described [13]. With single basepair resolution in Genebench, highly resolved PCR amplicon length was determined via migration of a standard size marker. Output data using Genemarker HID STR Human Identification Software, Version 1.51 (SoftGenetics LLC, State College, PA) shows fluorescent signal strength in RFU indicative of PCR sensitivity. Primers that gave fluorescence intensities of approximately 10^4^ RFUs of the specific product band without generating artifactual peaks were utilized for the multiplex assembly. The 10-plex panel was assembled by iterative addition of successful primers in a single 7 µL reaction. Peak signal balancing in the assembled multiplex assay was achieved by optimizing concentrations of the individual primers. Primers that caused signal inhibition of other loci were replaced with an alternative primer pair either by redesign or use of alternative target.

### Microfluidic Sanger Sequencing

PCR amplicons were subjected to microfluidic Sanger sequencing as previously described [61]. Singleplex PCR was carried out with similar template DNA amounts and thermal cycling conditions as in the multiplex PCR but using unlabeled primers for both amplification and sequencing. PCR products were directly used as template for microfluidic sequencing reactions using the BigDye® Terminator v3.1 cycle sequencing kit (Applied Biosystems, Foster City, CA) and the rapid thermal cycler and microfluidic PCR biochip as previously described [13]. For a given biothreat, a total of 20 independent Sanger sequencing reactions (10 forward and 10 reverse) were performed, with each reaction containing one sequencing primer (forward or reverse) to obtain bidirectional reads. Thermal cycling began with 60 seconds activation at 95°C, followed by 33 cycles (5 seconds at 95°C, 10 seconds at 50°C, and 40 seconds at 60°C) over approximately 32 minutes. Raw electropherograms were processed and basecalled using nnimbc4 (NNIM LLC, Salt Lake City, UT). Forward and reverse reads were aligned by importing the sequences in Codon Code Aligner (CodonCode Corporation, Dedham, MA).

### DNA Purification from Air Filter Media and Assessing PCR Inhibition

Air filter samples were washed in 5 mL sterile water with constant agitation for 5 minutes. Pelleted cells were lysed by sonication for 5 minutes using the VialTweeter sonicator (Hielscher USA, Inc.) followed by DNA purification as previously described [61]. To characterize presence of PCR inhibitors from the purified air filter DNA samples, PCR reactions containing 1 ng *B. subtilis* DNA and *B. subtilis* DNA spiked with air filter DNA as templates were prepared. A 16S rDNA gene universal bacterial primer was utilized and the expected amplicons analyzed on ethidium bromide-stained agarose gels. Percent PCR inhibition was calculated from the decrease in amplicon signal relative to unspiked *B. subtilis* control DNA.

## Supporting Information

Figure S1 Phylogram of experimentally obtained and retrieved *Ba, Bc* and *Bt* concatenated sequences after clustal alignment and visualization of the drawn PHYLIP tree with TreeView 1.6.6. Note that strains from which less than 4 loci were amplified were not included in the alignment/tree (i.e., *Bt* sv. *kurstaki* HD1, sv. *israelensis* 35646, *B. megaterium* and *B.* coagulans ATCC 7050). Also, strains included in the genotype table that have physical gaps of one or more *Ba* panel loci in the whole genome shotgun data (indicated with “#” in the table) have been omitted to avoid skewing of data (i.e., A2012, A0488, A0465, A0442, A0174, A0193, Tsiankovskii-I, A0389).
(TIFF)

Figure S2 Phylogram of experimentally obtained and retrieved *Ft* subspecies concatenated sequences after clustal alignment and visualization with PHYLIP (TreeView). Note that no *Fp* strains are included as only one of 10 loci was amplifiable with *Ft* panel primers.
(TIFF)

Figure S3 Sequence trace of *fop*A amplicon from focused sequencing of *Ft* 30-plex PCR template. Strain used was that of *Ft* WY96 with 1000 genome copies input to PCR. Aligned traces show the double-stranded coverage region (positions 55–255) of the 303 bp long *fop*A amplicon. A SNP distinguishing the *fop*A amplicon of strain WY96 ( = A) from that of the reference strain Schu S4 ( = G) is highlighted (black nucleotides). Both reads are of high quality as indicated by the Phred values (CodonCode Aligner assigns 255 and 254 bases of the forward and reverse reads, respectively, with Phred values of >20). Top trace is the forward read (sequenced using forward primer) and bottom trace is the reverse read (sequenced using reverse primer). Both amplicons are covered from end to end (5′ end of forward to 5′ end of reverse primer) albeit with single-stranded coverage for the first 55 and last 48 nucleotides (not shown).
(TIFF)

Figure S4 Phylogram of experimentally obtained and retrieved *Yp* and *Ypst* concatenated sequences after clustal alignment and visualization of the drawn PHYLIP (TreeView). Strains included in the genotype table that have physical gaps of one or more *Yp* panel loci in the whole genome shotgun data (indicated with “#” in the table) have been omitted to avoid skewing of data (i.e., IP275 and K1973002).
(TIFF)

Figure S5 Testing the *Yp* 10-plex assay against molar excesses of *Yp* near neighbors. *Yp* 10-plex panel against 10^5^ copies of *Ypst* YPIII DNA (left panel) and 1∶100 molar ratio of *Yp* Kim 10:*Ypst* (right panel). The length of the *Ypst arg*S amplicon is clearly distinguishable from the *Yp* amplicons. Two *arg*S specific products are observed for the spiked *Yp* DNA: one specific to *Ypst* YPIII at 395 bp and another specific for *Yp* KIM10 at 428 bp. When *Ypst* DNA is spiked in 100 fold excess to KIM10 DNA, the chromosomal targets of this strain are out-competing the targets of KIM10. All expected *Yp* KIM10 loci are still visible. The 3 red peaks (*glg*X, *arg*S, and *glp*D) and the 2 yellow peaks (*nuo*G and YPO1976) are due to incomplete color correction/bleedthrough from signal saturation (A). *Yp* 10-plex panel against 10^5^ copies of *Yk* CDC1457-B1 DNA (left panel) and 1∶100 molar ratio of *Yp* Kim 10:*Yk* (right panel). No specific products are amplified from *Yk*, as the primers were designed to be discriminatory against *Yk* loci based on available genome sequence data. A 100-fold molar excess of *Yk* DNA into *Yp* KIM10 DNA does not alter the KIM10 profile. The 3 small peaks in red are due to color pull-ups (B). *Yp* 10-plex panel against 10^5^ copies of *Yal* 670-B1 DNA (left panel) and 1∶100 molar ratio of *Yp* Kim 10:*Ya* (right panel). When *Yal* DNA is spiked in 100-fold molar excess to KIM10 DNA, all specific *Yp* loci are still clearly identifiable (C). *Yp* 10-plex panel against 10^5^ copies of *Ye* WA DNA (left panel) and 1∶100 molar ratio of *Yp* Kim 10:*Ye* (right panel). No *Yp* specific products are observed in *Ye* profile and addition of 100-fold molar excess of *Ye* DNA did not disturb the KIM10 profile. The 4 small peaks in red are due to color pull-ups (D).
(TIFF)

Figure S6 Sensitivity and specificity of the 30-plex PCR assay in the presence of molar excess of a representative environmental background strain. The profile from amplification of 10^6^ copies of *B. cepacia* 249 shows only background noise from EBS (A). Resulting profiles from simultaneous amplification of the 10 *Ba* loci with 100 copies of Sterne (B) and 10 *Yp* loci with 100 copies of Kim10 (C) in the presence of EBS (10^6^ copies of *B. cepacia* 249) are presented.
(TIFF)

Table S1 *Ba* target description, primer sequences, and 5′ fluorescent labels. Positions of amplicon boundaries based on Ames Ancestor chromosome and pXO1, AE017334.2 and AE017336.2, respectively, and amplicon lengths based on *in silico* range observed in *Ba* whole genome strains are also noted.
(DOCX)

Table S2 *Ft* target description, primer sequences, and 5′ fluorescent labels. Positions of amplicon boundaries based on Schu S4 chromosome, Genbank Acc# AJ7499949.2, and amplicon lengths based on *in silico* range observed in *Ft* whole genome strains are also noted.
(DOCX)

Table S3 *Yp* target description, primer sequences, and 5′ fluorescent labels. Positions of amplicon boundaries based on bv. *orientalis* CO92 chromosome, pMT, pPCP; Genbank Acc# AL590842.1, AL117211.1, AL109969.1, respectively, and amplicon lengths based on *in silico* range observed in *Yp* whole genome strains are also noted.
(DOCX)

## References

[1] Graham B, Talent JM, Allison GT (2008) World at risk: the report of the Commission on the Prevention of WMD Proliferation and Terrorism: Vintage.

[2] O'Toole T, Inglesby T (2009) Strategic priorities for US biosecurity. Biosecurity and Bioterrorism 7: 25–28. 19317617 10.1089/bsp.2009.1001

[3] Eubanks LM, Dickerson TJ, Janda KD (2007) Technological advancements for the detection of and protection against biological and chemical warfare agents. Chem Soc Rev 36: 458–470. 17325785 10.1039/b615227a

[4] Lim DV, Simpson JM, Kearns EA, Kramer MF (2005) Current and developing technologies for monitoring agents of bioterrorism and biowarfare. Clin Microbiol Rev. 583–607. 10.1128/CMR.18.4.583-607.2005 PMC1265906 16223949

[5] Bode E, Hurtle W, Norwood D (2004) Real-time PCR assay for a unique chromosomal sequence of Bacillus anthracis. Journal of Clinical Microbiology. 5825–5831. 10.1128/JCM.42.12.5825-5831.2004 PMC535252 15583318

[6] Larsson P, Svensson K, Karlsson L, Guala D, Granberg M, et al (2007) Canonical insertion-deletion markers for rapid DNA typing of Francisella tularensis. Emerging infectious diseases 13: 1725. 18217558 10.3201/eid1311.070603 PMC2874433

[7] Tomioka K, Peredelchuk M, Zhu X, Arena R, Volokhov D, et al (2005) A multiplex polymerase chain reaction microarray assay to detect bioterror pathogens in blood. The Journal of molecular diagnostics: JMD 7: 486. 16237218 10.1016/S1525-1578(10)60579-X PMC1888491

[8] Tomaso H, Reisinger EC, Al Dahouk S, Frangoulidis D, Rakin A, et al (2003) Rapid detection of Yersinia pestis with multiplex real-time PCR assays using fluorescent hybridisation probes. FEMS Immunology and Medical Microbiology 38: 117–126. 13129646 10.1016/S0928-8244(03)00184-6

[9] Stewart A, Satterfield B, Cohen M, O'Neill K, Robison R (2008) A quadruplex real-time PCR assay for the detection of Yersinia pestis and its plasmids. Journal of medical microbiology 57: 324–331. 18287295 10.1099/jmm.0.47485-0

[10] Ingmar J, Raditijo H, Jasper B (2010) Reliable detection of Bacillus anthracis, Francisella tularensis and Yersinia pestis by using multiplex qPCR including internal controls for nucleic acid extraction and amplification. 10.1186/1471-2180-10-314 PMC3016324 21143837

[11] Janse I, Bok JM, Hamidjaja RA, Hodemaekers HM, van Rotterdam BJ (2012) Development and Comparison of Two Assay Formats for Parallel Detection of Four Biothreat Pathogens by Using Suspension Microarrays. PLoS One 7: e31958. 22355407 10.1371/journal.pone.0031958 PMC3280232

[12] Wilson WJ, Erler AM, Nasarabadi SL, Skowronski EW, Imbro PM (2005) A multiplexed PCR-coupled liquid bead array for the simultaneous detection of four biothreat agents. Mol Cell Probes. 137–144. 10.1016/j.mcp.2004.10.005 15680215

[13] Giese H, Lam R, Selden R, Tan E (2009) Fast multiplexed polymerase chain reaction for conventional and microfluidic short tandem repeat analysis. J Forensic Sci 54: 1287–1296. 19840207 10.1111/j.1556-4029.2009.01200.x

[14] Schumm J, Gutierrez-Mateo C, Tan E, Selden RF (2012) A 27-Locus STR Assay to Meet All United States and European Law Enforcement Agency Standards*. Journal of Forensic Science. In Press. 10.1111/1556-4029.12214 23822765

[15] Harrell LJ, Andersen GL, Wilson KH (1995) Genetic variability of Bacillus anthracis and related species. Journal of clinical microbiology 33: 1847. 7665658 10.1128/jcm.33.7.1847-1850.1995 PMC228283

[16] Keim P, Gruendike JM, Klevytska AM, Schupp JM, Challacombe J, et al (2009) The genome and variation of Bacillus anthracis. Molecular aspects of medicine 30: 397–405. 19729033 10.1016/j.mam.2009.08.005 PMC3034159

[17] Price LB, Hugh-Jones M, Jackson PJ, Keim P (1999) Genetic diversity in the protective antigen gene of Bacillus anthracis. Journal of bacteriology 181: 2358. 10197996 10.1128/jb.181.8.2358-2362.1999 PMC93658

[18] Jensen G, Hansen B, Eilenberg J, Mahillon J (2003) The hidden lifestyles of Bacillus cereus and relatives. Environmental Microbiology 5: 631–640. 12871230 10.1046/j.1462-2920.2003.00461.x

[19] Helgason E, Okstad OA, Caugant DA, Johansen HA, Fouet A, et al (2000) Bacillus anthracis, Bacillus cereus, and Bacillus thuringiensis–one species on the basis of genetic evidence. Applied and Environmental Microbiology 66: 2627–2630. 10831447 10.1128/aem.66.6.2627-2630.2000 PMC110590

[20] Bavykin SG, Lysov YP, Zakhariev V, Kelly JJ, Jackman J, et al (2004) Use of 16S rRNA, 23S rRNA, and gyrB gene sequence analysis to determine phylogenetic relationships of Bacillus cereus group microorganisms. Journal of clinical microbiology 42: 3711. 15297521 10.1128/JCM.42.8.3711-3730.2004 PMC497648

[21] Helgason E, Tourasse NJ, Meisal R, Caugant DA, Kolsto AB (2004) Multilocus sequence typing scheme for bacteria of the Bacillus cereus group. Applied and Environmental Microbiology 70: 191–201. 14711642 10.1128/AEM.70.1.191-201.2004 PMC321270

[22] Daffonchio D, Raddadi N, Merabishvili M, Cherif A, Carmagnola L, et al (2006) Strategy for identification of Bacillus cereus and Bacillus thuringiensis strains closely related to Bacillus anthracis. Applied and Environmental Microbiology 72: 1295. 16461679 10.1128/AEM.72.2.1295-1301.2006 PMC1392923

[23] Keim P, Kalif A, Schupp J, Hill K, Travis SE, et al (1997) Molecular evolution and diversity in Bacillus anthracis as detected by amplified fragment length polymorphism markers. Journal of bacteriology 179: 818. 9006038 10.1128/jb.179.3.818-824.1997 PMC178765

[24] Jackson PJ, Hill K, Laker M, Ticknor L, Keim P (1999) Genetic comparison of Bacillus anthracis and its close relatives using amplified fragment length polymorphism and polymerase chain reaction analysis. Journal of applied microbiology 87: 263–269. 10475963 10.1046/j.1365-2672.1999.00884.x

[25] Lista F, Faggioni G, Valjevac S, Ciammaruconi A, Vaissaire J, et al (2006) Genotyping of Bacillus anthracis strains based on automated capillary 25-loci multiple locus variable-number tandem repeats analysis. BMC microbiology 6: 33. 16600037 10.1186/1471-2180-6-33 PMC1479350

[26] Sjostedt A (2003) Family XVII, Francisellaceae. Genus I, Francisella. Bergey's Manual of Systematic Bacteriology. New York, NY: Springer-Verlag. 111–113.

[27] Cross J, Penn R (2000) Francisella tularensis (tularemia). Principles and Practice of Infectious Diseases Philadelphia, Pa: Churchill Livingstone. 2393–2402.

[28] Nubel U, Reissbrodt R, Weller A, Grunow R, Porsch-Ozcurumez M, et al (2006) Population structure of Francisella tularensis. Journal of bacteriology 188: 5319. 16816208 10.1128/JB.01662-05 PMC1539956

[29] Forsman M, Sandstrom G, Sjostedt A (1994) Analysis of 16S ribosomal DNA sequences of Francisella strains and utilization for determination of the phylogeny of the genus and for identification of strains by PCR. International journal of systematic bacteriology 44: 38–46. 8123561 10.1099/00207713-44-1-38

[30] Splettstoesser W, Seibold E, Zeman E, Trebesius K, Podbielski A (2010) Rapid differentiation of Francisella species and subspecies by fluorescent in situ hybridization targeting the 23S rRNA. BMC microbiology 10: 72. 20205957 10.1186/1471-2180-10-72 PMC2844405

[31] Svensson K, Larsson P, Johansson D, Bystrom M, Forsman M, et al (2005) Evolution of subspecies of Francisella tularensis. Journal of bacteriology 187: 3903. 15901721 10.1128/JB.187.11.3903-3908.2005 PMC1112057

[32] La Scola B, Elkarkouri K, Li W, Wahab T, Fournous G, et al (2008) Rapid comparative genomic analysis for clinical microbiology: the Francisella tularensis paradigm. Genome research 18: 742–750. 18407970 10.1101/gr.071266.107 PMC2336804

[33] Farlow J, Smith KL, Wong J, Abrams M, Lytle M, et al (2001) Francisella tularensis strain typing using multiple-locus, variable-number tandem repeat analysis. Journal of clinical microbiology 39: 3186. 11526148 10.1128/JCM.39.9.3186-3192.2001 PMC88316

[34] Johansson A, Goransson I, Larsson P, Sjostedt A (2001) Extensive allelic variation among Francisella tularensis strains in a short-sequence tandem repeat region. Journal of clinical microbiology 39: 3140. 11526142 10.1128/JCM.39.9.3140-3146.2001 PMC88310

[35] Johansson A, Farlow J, Larsson P, Dukerich M, Chambers E, et al (2004) Worldwide genetic relationships among Francisella tularensis isolates determined by multiple-locus variable-number tandem repeat analysis. Journal of bacteriology 186: 5808–5818. 15317786 10.1128/JB.186.17.5808-5818.2004 PMC516809

[36] Achtman M, Zurth K, Morelli G, Torrea G, Guiyoule A, et al (1999) Yersinia pestis, the cause of plague, is a recently emerged clone of Yersinia pseudotuberculosis. Proceedings of the National Academy of Sciences 96: 14043. 10.1073/pnas.96.24.14043 PMC24187 10570195

[37] Achtman M, Morelli G, Zhu P, Wirth T, Diehl I, et al (2004) Microevolution and history of the plague bacillus, Yersinia pestis. Proceedings of the National Academy of Sciences of the United States of America 101: 17837. 15598742 10.1073/pnas.0408026101 PMC535704

[38] Trebesius K, Harmsen D, Rakin A, Schmelz J, Heesemann J (1998) Development of rRNA-Targeted PCR and In Situ Hybridization with Fluorescently Labelled Oligonucleotides for Detection of Yersinia Species. Journal of Clinical Microbiology 36: 2557–2564. 9705392 10.1128/jcm.36.9.2557-2564.1998 PMC105162

[39] Adair D, Worsham P, Hill K, Klevytska A, Jackson P, et al (2000) Diversity in a variable-number tandem repeat from Yersinia pestis. Journal of clinical microbiology 38: 1516. 10747136 10.1128/jcm.38.4.1516-1519.2000 PMC86479

[40] Girard JM, Wagner DM, Vogler AJ, Keys C, Allender CJ, et al (2004) Differential plague-transmission dynamics determine Yersinia pestis population genetic structure on local, regional, and global scales. Proceedings of the National Academy of Sciences of the United States of America 101: 8408. 15173603 10.1073/pnas.0401561101 PMC420407

[41] Drancourt M, Roux V, Vu Dang L, Tran-Hung L, Castex D, et al (2004) Genotyping, Orientalis-like Yersinia pestis, and plague pandemics. Emerging infectious diseases 10: 1585–1592. 15498160 10.3201/eid1009.030933 PMC3320270

[42] Motin VL, Georgescu AM, Elliott JM, Hu P, Worsham PL, et al (2002) Genetic variability of Yersinia pestis isolates as predicted by PCR-based IS100 genotyping and analysis of structural genes encoding glycerol-3-phosphate dehydrogenase (glpD). Journal of bacteriology 184: 1019. 11807062 10.1128/jb.184.4.1019-1027.2002 PMC134790

[43] Pannucci J, Okinaka RT, Sabin R, Kuske CR (2002) Bacillus anthracis pXO1 plasmid sequence conservation among closely related bacterial species. Journal of bacteriology 184: 134. 11741853 10.1128/JB.184.1.134-141.2002 PMC134754

[44] Pannucci J, Okinaka R, Williams E, Sabin R, Ticknor L, et al (2002) DNA sequence conservation between the Bacillus anthracis pXO2 plasmid and genomic sequence from closely related bacteria. BMC genomics 3: 34. 12473162 10.1186/1471-2164-3-34 PMC140023

[45] Hoffmaster AR, Ravel J, Rasko DA, Chapman GD, Chute MD, et al (2004) Identification of anthrax toxin genes in a Bacillus cereus associated with an illness resembling inhalation anthrax. Proc Natl Acad Sci U S A 101: 8449–8454. 15155910 10.1073/pnas.0402414101 PMC420414

[46] Klee SR, Brzuszkiewicz EB, Nattermann H, Bruggemann H, Dupke S, et al (2010) The genome of a Bacillus isolate causing anthrax in chimpanzees combines chromosomal properties of B. cereus with B. anthracis virulence plasmids. PLoS One 5: e10986. 20634886 10.1371/journal.pone.0010986 PMC2901330

[47] Rasko DA, Altherr MR, Han CS, Ravel J (2005) Genomics of the Bacillus cereus group of organisms. FEMS microbiology reviews 29: 303–329. 15808746 10.1016/j.femsre.2004.12.005

[48] Green B, Battisti L, Koehler T, Thorne C, Ivins B (1985) Demonstration of a capsule plasmid in Bacillus anthracis. Infection and immunity 49: 291–297. 3926644 10.1128/iai.49.2.291-297.1985 PMC262013

[49] Read TD, Peterson SN, Tourasse N, Baillie LW, Paulsen IT, et al (2003) The genome sequence of Bacillus anthracis Ames and comparison to closely related bacteria. Nature 423: 81–86. 12721629 10.1038/nature01586

[50] CDC.gov, Centers for Disease Control and Prevention. Anthrax Sterne strain (34F2) of Bacillus anthracis. National Center for Emerging and Zoonotic Infectious Diseases CDC. Available: http://www.cdc.gov/nczved/divisions/dfbmd/diseases/anthrax_sterne/. Accessed 2012 Oct.

[51] Page R, Division of Ecology and Evolutionary Biology. Available: http://taxonomy.zoology.gla.ac.uk/rod/rod/html. Accessed 2012 Apr.

[52] Ludu JS, de Bruin OM, Duplantis BN, Schmerk CL, Chou AY, et al.. (2008) The Francisella pathogenicity island protein PdpD is required for full virulence and associates with homologues of the type VI secretion system. J Bacteriol. 4584–4595. 10.1128/JB.00198-08 PMC2446798 18469101

[53] Pandya G, Holmes M, Petersen J, Pradhan S, Karamycheva S, et al (2009) Whole genome single nucleotide polymorphism based phylogeny of Francisella tularensis and its application to the development of a strain typing assay. BMC microbiology 9: 213. 19811647 10.1186/1471-2180-9-213 PMC2767358

[54] Huang XZ, Nikolich MP, Lindler LE (2006) Current trends in plague research: from genomics to virulence. Clinical medicine & research 4: 189–199. 16988099 10.3121/cmr.4.3.189 PMC1570480

[55] Bhaduri S, Sommers CH (2008) Detection of Yersinia Pestis by Comparison of Virulence Plasmid (PYV/PCD) and Associated Phenotypes in Yersinia Species. Journal of Food Safety 28: 453–466.

[56] Shendure J, Ji H (2008) Next-generation DNA sequencing. Nature biotechnology 26: 1135–1145. 10.1038/nbt1486 18846087

[57] Voelkerding KV, Dames SA, Durtschi JD (2009) Next-generation sequencing: from basic research to diagnostics. Clinical chemistry 55: 641–658. 19246620 10.1373/clinchem.2008.112789

[58] Stapley J, Reger J, Feulner PGD, Smadja C, Galindo J, et al (2010) Adaptation genomics: the next generation. Trends in Ecology & Evolution 25: 705–712. 20952088 10.1016/j.tree.2010.09.002

[59] Altschul SF, Gish W, Miller W, Myers EW, Lipman DJ (1990) Basic local alignment search tool. Journal of molecular biology 215: 403–410. 2231712 10.1016/S0022-2836(05)80360-2

[60] Larkin MA, Blackshields G, Brown NP, Chenna R, McGettigan PA, et al (2007) Clustal W and Clustal X version 2.0. Bioinformatics 23: 2947. 17846036 10.1093/bioinformatics/btm404

[61] Read TD, Turingan RS, Cook C, Giese H, Thomann UH, et al (2010) Rapid multi-locus sequence typing using microfluidic biochips. PLoS One 5: e10595. 20485679 10.1371/journal.pone.0010595 PMC2868872

