# Supplementary figures and images for: Rapid Focused Sequencing: A Multiplexed Assay for Simultaneous Detection and Strain Typing of Bacillus anthracis, Francisella tularensis, and Yersinia pestis
Source: PLoS One. 2013 Feb 13;8(2):e56093. doi: 10.1371/journal.pone.0056093 (PMC3572037; doi:10.1371/journal.pone.0056093)

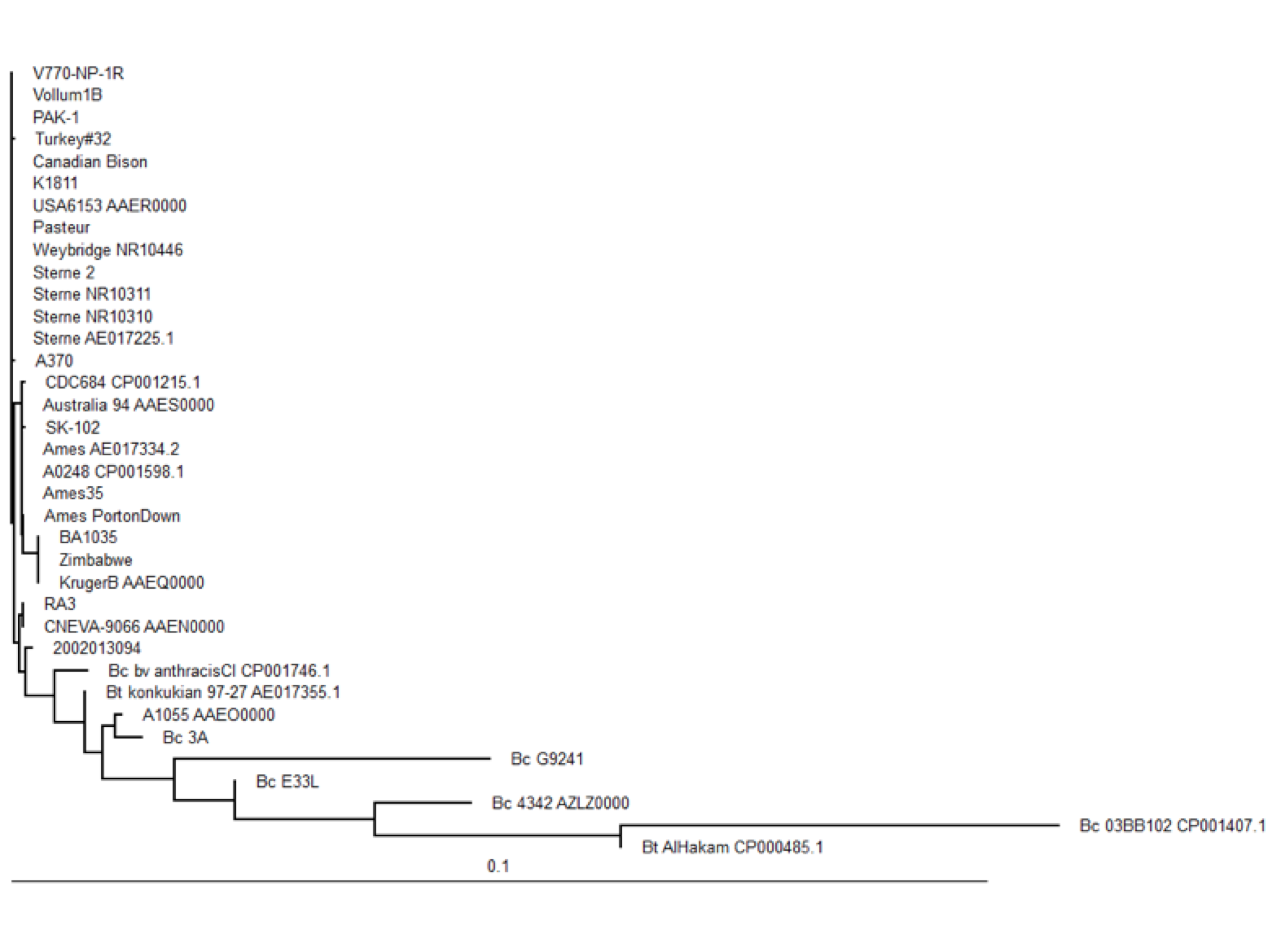

Supplement: Figure S1 — Phylogram of experimentally obtained and retrieved Ba, Bc and Bt concatenated sequences after clustal alignment and visualization of the drawn PHYLIP tree with TreeView 1.6.6. Note that strains from which less than 4 loci were amplified were not included in the alignment/tree (i.e., Bt sv. kurstaki HD1, sv. israelensis 35646, B. megaterium and B. coagulans ATCC 7050). Also, strains included in the genotype table that have physical gaps of one or more Ba panel loci in the whole genome shotgun data (indicated with “#” in the table) have been omitted to avoid skewing of data (i.e., A2012, A0488, A0465, A0442, A0174, A0193, Tsiankovskii-I, A0389). (TIFF) [file pone.0056093.s001.tiff]

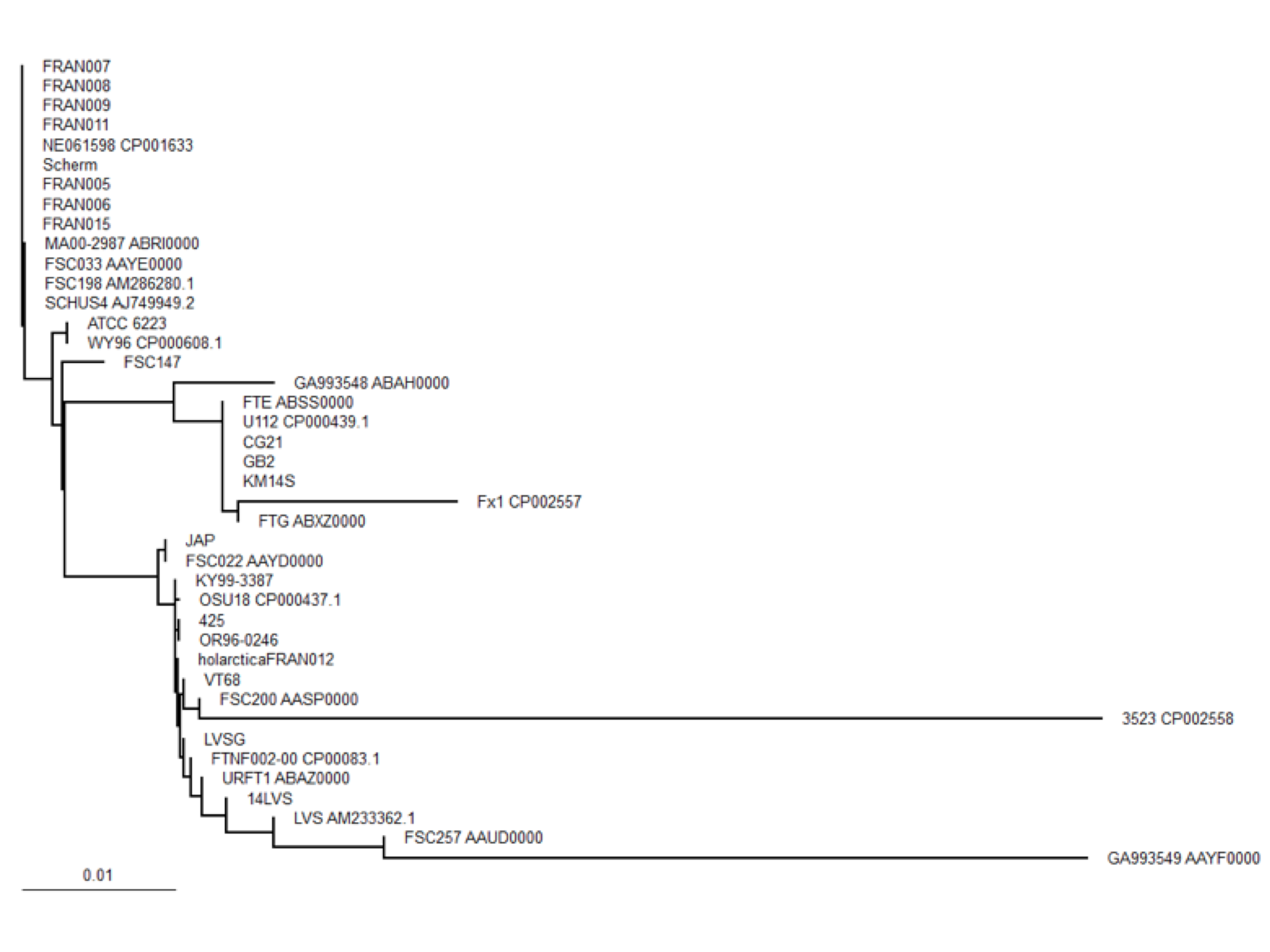

Supplement: Figure S2 — Phylogram of experimentally obtained and retrieved Ft subspecies concatenated sequences after clustal alignment and visualization with PHYLIP (TreeView). Note that no Fp strains are included as only one of 10 loci was amplifiable with Ft panel primers. (TIFF) [file pone.0056093.s002.tiff]

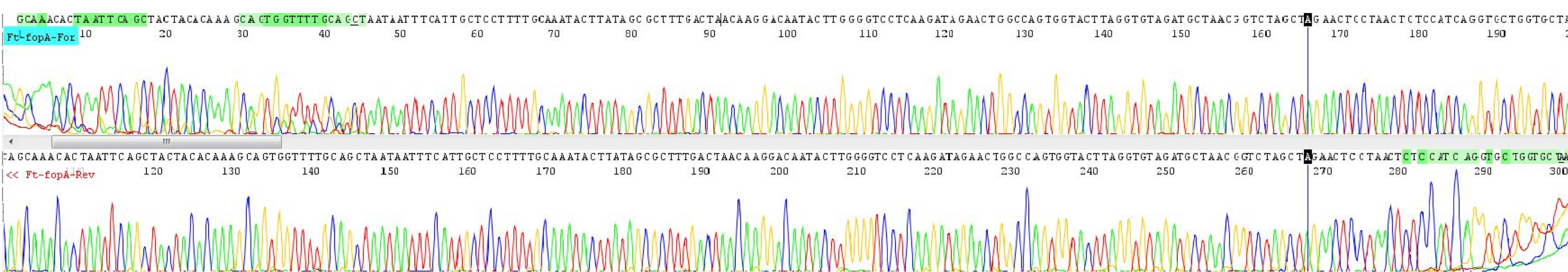

Supplement: Figure S3 — Sequence trace of fopA amplicon from focused sequencing of Ft 30-plex PCR template. Strain used was that of Ft WY96 with 1000 genome copies input to PCR. Aligned traces show the double-stranded coverage region (positions 55–255) of the 303 bp long fopA amplicon. A SNP distinguishing the fopA amplicon of strain WY96 ( = A) from that of the reference strain Schu S4 ( = G) is highlighted (black nucleotides). Both reads are of high quality as indicated by the Phred values (CodonCode Aligner assigns 255 and 254 bases of the forward and reverse reads, respectively, with Phred values of >20). Top trace is the forward read (sequenced using forward primer) and bottom trace is the reverse read (sequenced using reverse primer). Both amplicons are covered from end to end (5′ end of forward to 5′ end of reverse primer) albeit with single-stranded coverage for the first 55 and last 48 nucleotides (not shown). (TIFF) [file pone.0056093.s003.tiff]

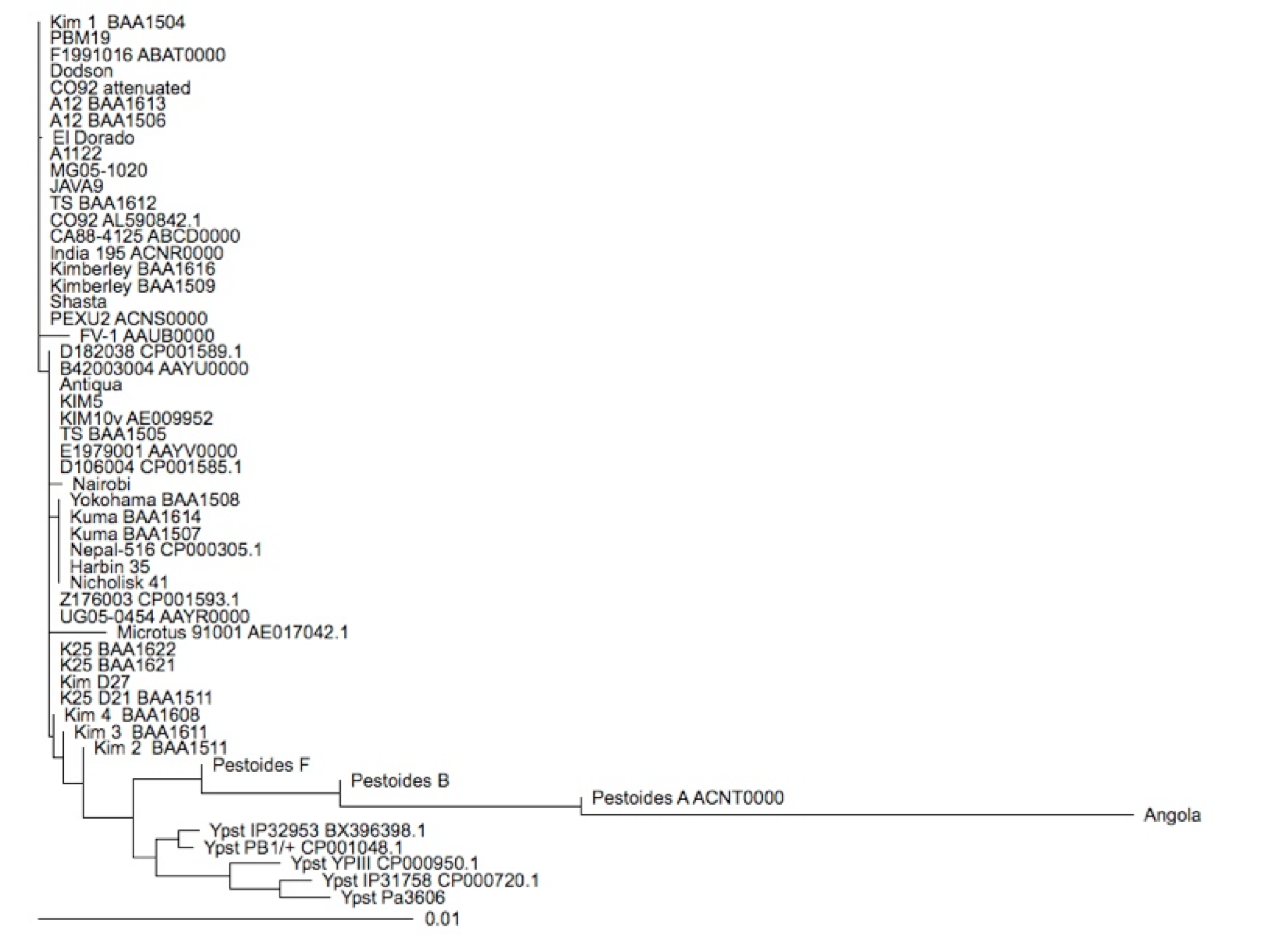

Supplement: Figure S4 — Phylogram of experimentally obtained and retrieved Yp and Ypst concatenated sequences after clustal alignment and visualization of the drawn PHYLIP (TreeView). Strains included in the genotype table that have physical gaps of one or more Yp panel loci in the whole genome shotgun data (indicated with “#” in the table) have been omitted to avoid skewing of data (i.e., IP275 and K1973002). (TIFF) [file pone.0056093.s004.tiff]

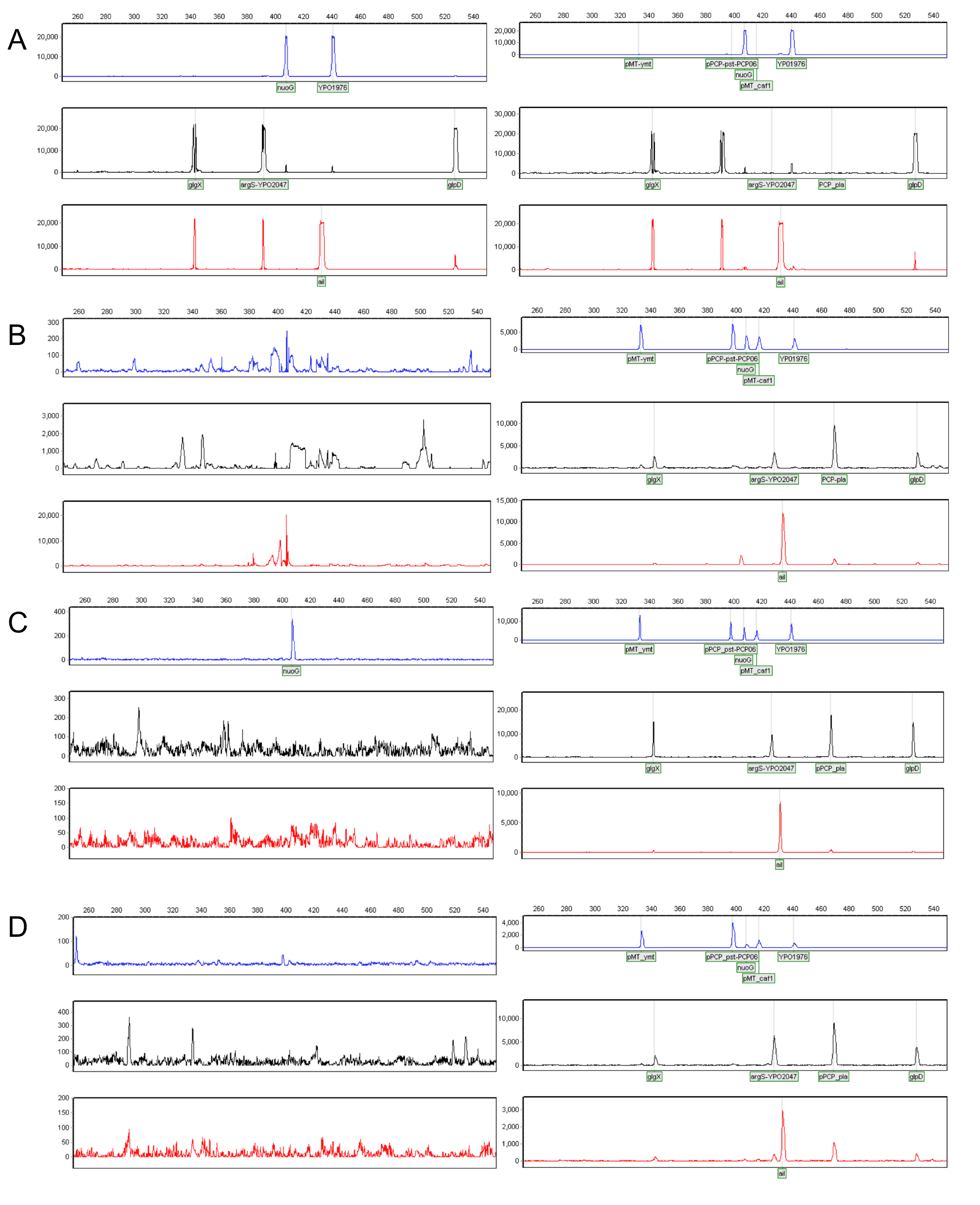

Supplement: Figure S5 — Testing the Yp 10-plex assay against molar excesses of Yp near neighbors. Yp 10-plex panel against 105 copies of Ypst YPIII DNA (left panel) and 1∶100 molar ratio of Yp Kim 10:Ypst (right panel). The length of the Ypst argS amplicon is clearly distinguishable from the Yp amplicons. Two argS specific products are observed for the spiked Yp DNA: one specific to Ypst YPIII at 395 bp and another specific for Yp KIM10 at 428 bp. When Ypst DNA is spiked in 100 fold excess to KIM10 DNA, the chromosomal targets of this strain are out-competing the targets of KIM10. All expected Yp KIM10 loci are still visible. The 3 red peaks (glgX, argS, and glpD) and the 2 yellow peaks (nuoG and YPO1976) are due to incomplete color correction/bleedthrough from signal saturation (A). Yp 10-plex panel against 105 copies of Yk CDC1457-B1 DNA (left panel) and 1∶100 molar ratio of Yp Kim 10:Yk (right panel). No specific products are amplified from Yk, as the primers were designed to be discriminatory against Yk loci based on available genome sequence data. A 100-fold molar excess of Yk DNA into Yp KIM10 DNA does not alter the KIM10 profile. The 3 small peaks in red are due to color pull-ups (B). Yp 10-plex panel against 105 copies of Yal 670-B1 DNA (left panel) and 1∶100 molar ratio of Yp Kim 10:Ya (right panel). When Yal DNA is spiked in 100-fold molar excess to KIM10 DNA, all specific Yp loci are still clearly identifiable (C). Yp 10-plex panel against 105 copies of Ye WA DNA (left panel) and 1∶100 molar ratio of Yp Kim 10:Ye (right panel). No Yp specific products are observed in Ye profile and addition of 100-fold molar excess of Ye DNA did not disturb the KIM10 profile. The 4 small peaks in red are due to color pull-ups (D). (TIFF) [file pone.0056093.s005.tiff]

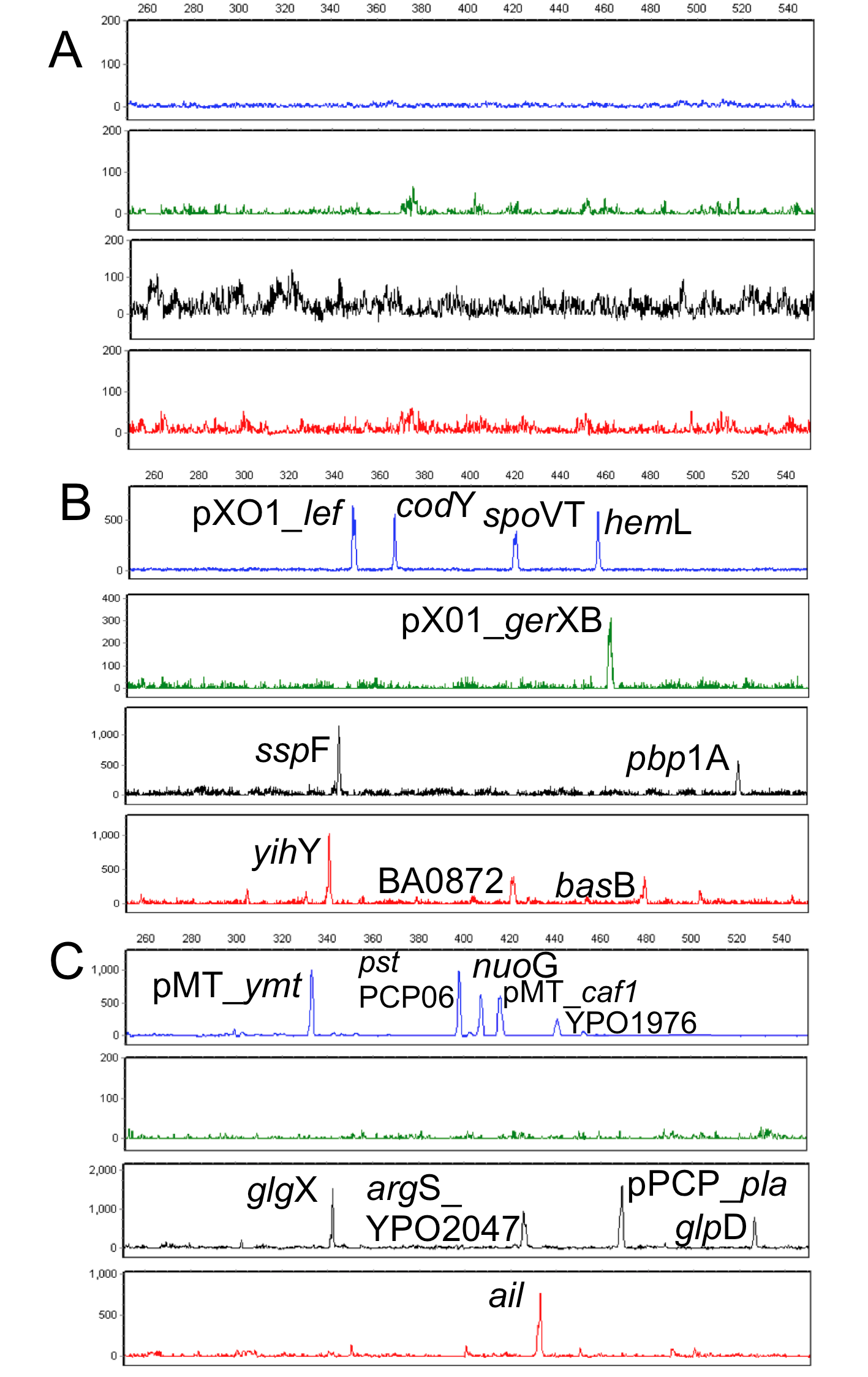

Supplement: Figure S6 — Sensitivity and specificity of the 30-plex PCR assay in the presence of molar excess of a representative environmental background strain. The profile from amplification of 106 copies of B. cepacia 249 shows only background noise from EBS (A). Resulting profiles from simultaneous amplification of the 10 Ba loci with 100 copies of Sterne (B) and 10 Yp loci with 100 copies of Kim10 (C) in the presence of EBS (106 copies of B. cepacia 249) are presented. (TIFF) [file pone.0056093.s006.tiff]
