# Supplementary material for: Rapid Focused Sequencing: A Multiplexed Assay for Simultaneous Detection and Strain Typing of Bacillus anthracis, Francisella tularensis, and Yersinia pestis
Source: PLoS One. 2013 Feb 13;8(2):e56093. doi: 10.1371/journal.pone.0056093 (PMC3572037; doi:10.1371/journal.pone.0056093)
Supplement: Table S1 — Ba target description, primer sequences, and 5′ fluorescent labels. Positions of amplicon boundaries based on Ames Ancestor chromosome and pXO1, AE017334.2 and AE017336.2, respectively, and amplicon lengths based on in silico range observed in Ba whole genome strains are also noted. (DOCX) [file pone.0056093.s007.docx]

**Table S1.** ***Ba* target description, primer sequences, and 5’ fluorescent labels.**

| ***Ba* Targets** | **Position Ames Ancestor** | **Forward Primer/Reverse Primer** | **Amplicon Size (bp)** | **Gene Category: Function** |
| --- | --- | --- | --- | --- |
| pXO1*_lef* | 151,111-150,762 | FAM-CAGCTTAAGGAACATCCCACAGAC/ TGAATTATGTCATCTTTCTTTGGCTCA | 350 | Virulence: pXO1-encoded virulence factor (lethal factor) |
| pXO1*_ger*XB | 137,700-138,160 | JOE-AAGGGATGATTTATCCAGCCGC/ TGCTGAAATGGGTAAGAAAACATGAG | 461 | Sporulation: pXO1-encoded spore germination response protein, *B. subtilis* *ger*XB homologue [[1](#_ENREF_1), [2](#_ENREF_2)] |
| *ssp*F | 47,540-47,881 | TMR-GTTGCAACGTGTGTCGTATAGC/ GCTGTTCTGCAATTTCTATAGCACG | 342 | Sporulation: small-acid soluble spore protein F, *B. cereus* homologue [[3](#_ENREF_3)], conserved in *Ba* |
| *spo*VT | 58,273-58,694 | FAM-TCGAGAAGGGGACCCATTAGAA/ CAGCAGTATTGACCGCTTTGTG | 422 | Sporulation: stage V sporulation protein T, transcriptional regulator AbrB homologue [[4](#_ENREF_4)], conserved in *Ba* |
| GBAA0872 | 881,262-880,841 | ROX-TTCTTACTGGCCAAACCGTCTT/ TTCGATTTAAAAATGCGGCGGA | 422 | Metabolism: N-acetylmuramoyl-L-alanine amidase [[5](#_ENREF_5)], cell wall peptidoglycan metabolism enzyme, highly conserved protein in *Ba* |
| *hem*L | 4,269,357-4,268,901 | ACTTGTTTAGGTAAAGTAATYGGTGGT/ FAM-TCGCTTCAATATCAGCATCACTATGTA | 457 | Metabolism: glutamate -1-semialdehyde-2,1-aminomutase (key enzyme in heme biosysnthesis pathway) [[6](#_ENREF_6)] |
| *bas*B, GBAA0871 | 880,178-880,666 | ROX-TCAAGCGCCAGAAGGTTATGAGTT/ GGAAGCTGTTGAGCATGAGAGGTA | 385-615 | Metabolism: LPXTG-motif containing surface-cell wall protein, sortase A substrate [[7](#_ENREF_7)], gene harbors internal VNTR-like repeat [[8](#_ENREF_8)]. |
| *pbp*1A | 2,179,564-2,180,018 | TMR-GAGCGTACCGGATACAACAGAA/ CTTATTGACCATTATTAGCAGGGGC | 456-533 | Metabolism: penicillin-binding protein 1A, cell-wall synthesis enzyme, gene harbors internal VNTR-like repeat[[8](#_ENREF_8)] |
| *yih*Y-GBAA0410 | 428,604-428,942 | ROX-CTTGGCGGCGAAATTAATGGTT/ TTCCATTCGGTGTTATTGGGCA | 339-340 | Metabolism: Ribonuclease BN homologue YihY and ATPase (GBAA0410), amplicon spans 3’ ends of ORFs and intergenic region (IGS), highly conserved in *Ba* |
| *cod*Y | 3,643,422-3,643,057 | FAM-TAAAATTGCTGACCGCGTAGGA/ GTCGAAAATGCGAACAATGTAAAACT | 366-367 | Virulence: regulatory protein, required for toxin gene expression and full virulence[[9](#_ENREF_9)], amplicon spans 3’ end of *cod*Y and adjacent IGS containing indels. |

Positions of amplicon boundaries based on Ames Ancestor chromosome and pXO1, AE017334.2 and AE017336.2, respectively, and amplicon lengths based on *in silico* range observed in *Ba* whole genome strains are also noted.

**References**

1. Okinaka R, Cloud K, Hampton O, Hoffmaster A, Hill K, et al. (1999) Sequence and organization of pXO1, the large Bacillus anthracis plasmid harboring the anthrax toxin genes. Journal of bacteriology 181: 6509.

2. Ross C, Abel-Santos E (2010) The Ger receptor family from sporulating bacteria. Current issues in molecular biology 12: 147.

3. Loshon CA, Beary KE, Chander M, Setlow P (1994) Cloning and sequencing of the sspF (originally 0.3 kb) genes from Bacillus cereus and Bacillus megaterium. Gene 150: 203-204.

4. Dong TC, Cutting SM, Lewis RJ (2004) DNA binding studies on the Bacillus subtilis transcriptional regulator and AbrB homologue, SpoVT. FEMS microbiology letters 233: 247-256.

5. Ravel J, Jiang L, Stanley ST, Wilson MR, Decker RS, et al. (2009) The complete genome sequence of Bacillus anthracis Ames" Ancestor". Journal of bacteriology 191: 445.

6. Jahn D, Verkamp E (1992) Glutamyl-transfer RNA: a precursor of heme and chlorophyll biosynthesis. Trends in biochemical sciences 17: 215-218.

7. Aucher W, Davison S, Fouet A (2011) Characterization of the Sortase Repertoire in Bacillus anthracis. PLoS One 6: e27411.

8. Le Fleche P, Hauck Y, Onteniente L, Prieur A, Denoeud F, et al. (2001) A tandem repeats database for bacterial genomes: application to the genotyping of Yersinia pestis and Bacillus anthracis. BMC microbiology 1: 2.

9. Van Schaik W, Chateau A, Dillies MA, Coppee JY, Sonenshein AL, et al. (2009) The global regulator CodY regulates toxin gene expression in Bacillus anthracis and is required for full virulence. Infection and immunity 77: 4437.
