# Supplementary material for: Rapid Focused Sequencing: A Multiplexed Assay for Simultaneous Detection and Strain Typing of Bacillus anthracis, Francisella tularensis, and Yersinia pestis
Source: PLoS One. 2013 Feb 13;8(2):e56093. doi: 10.1371/journal.pone.0056093 (PMC3572037; doi:10.1371/journal.pone.0056093)
Supplement: Table S2 — Ft target description, primer sequences, and 5′ fluorescent labels. Positions of amplicon boundaries based on Schu S4 chromosome, Genbank Acc# AJ7499949.2, and amplicon lengths based on in silico range observed in Ft whole genome strains are also noted. (DOCX) [file pone.0056093.s008.docx]

**Table S2*. Ft* target description, primer sequences, and 5’ fluorescent labels.**

| ***Ft* Targets** | **Position SchuS4** | **Forward Primer/Reverse Primer** | **Amplicon Size [bp]** | **Gene Category: Function** |
| --- | --- | --- | --- | --- |
| FPI_ *igl*C | 1,399,453-1,399,144 and 1,792,788-1,792,479 | ROX-AACCCCTTTAGAAATGGCGAG/ ATTTTTATCAAGTTTCTCCCCAGC | 353 | Virulence: FPI-encoded 23kD intracellular growth protein essential for phagosome-escape; part of type VI secretion system [[1](#_ENREF_1), [2](#_ENREF_2)] |
| *fop*A | 599,361-599,663 | TMR-TTAGGTTCAGCTACAGCATCTAT/ GTTGTACTGTACAGCGAAGTATTTAT | 302 | Virulence: outer-membrane protein, protective antigen [[3](#_ENREF_3), [4](#_ENREF_4)] |
| FTT0900-*tul*4 | 909,673-910,169 | TMR-CATCTTGATCTTATCTTAGCGACT/ AACTTACTTTTGCAGTTGGCTT | 464-498 | Virulence: intergenic region between 17kD lipoprotein/major antigen gene tul4 [[5](#_ENREF_5)] and 13kD membrane protein gene FTT0900. |
| FPI_ *pdp*D | 1,404,127-1,403,818  and 1,797,462-1,797,153 | ROX-AGAGAATATACATACCCCGAAATTGA/ ATAAGTTTGGTATCTTAAAAGGAGACT | 361 | Virulence: FPI-encoded type VI secretion system protein, which is necessary for full virulence [[6](#_ENREF_6)]; deletion of locus in *holarctica* (type B) strains may explain reduced pathogenicity as compared to type A strains [[7](#_ENREF_7)]. |
| *acp*A | 239,157-139,417 | FAM-CGATATTAAAGGAAGACAAGGCTAT/ ATCCACTACTAATCCTGTCTTAGG | 261 | Virulence: acid phosphatase A, which aids survival in macrophages by inhibition of respiratory bursts [[8](#_ENREF_8)] but maybe not essential for virulence in type A strains [[9](#_ENREF_9)] |
| *pep*O | 1,229,426-1,299,123 | FAM-AACTCATCTCAAATGATTGTGAGTA/ ATAATGATAAACTTCTCTGTTTCTGG | 307-353 | Metabolism/Virulence: secreted protease PepO. Secretion is inhibited by a mutation in type A and B strains [[10](#_ENREF_10)] |
| *mig*R | 712,737-713,174 | FAM-GGGTAGGATTGGTATTAAGTATATCG/ CCGACATTATCCTCTTGCTGG | 436 | Gene Expression/Virulence: MigR is a transacting regulatory protein involved in *igl*ABCD gene operon expression [[11](#_ENREF_11)] |
| *gyr* | 1,442,997-1,442,672 | GGTTTTTATATTTCATAACCGTGTTG/ TMR-CTTATCTGTTTTTGGTGAACTGC | 325-409 | Metabolism: ORF FTT1395c encodes ATP binding subunit/regulatory subunit of a DNA helicase (*gyr*) |
| *spe*A | 447,282-447,770 | FAM-CAGATATTGAGAATGAAATCCTACAT/ ATTTGACTGTATCCTCAGAGAGT | 484**-**494 | Metabolism: ORF FTT0423 encodes an arginine decarboxylase |
| FTT0082 | 85,005-85,391 | ROX-ACGAGCTTTTATTGATTTTATTTCCC/ CTTATATGCTTTAATGAAGGAGTCAA | 381-387 | Gene Expression: FTT0082 may be a pseudogene in certain *Ft* strains. It encodes a putative LysR family transcriptional regulator. |

Positions of amplicon boundaries based on Schu S4 chromosome, Genbank Acc# AJ7499949.2, and amplicon lengths based on *in silico* range observed in *Ft* whole genome strains are also noted.

**References**

1. Santic M, Molmeret M, Klose KE, Jones S, Kwaik YA (2005) The Francisella tularensis pathogenicity island protein IglC and its regulator MglA are essential for modulating phagosome biogenesis and subsequent bacterial escape into the cytoplasm. Cellular microbiology 7: 969-979.

2. de Bruin OM, Duplantis BN, Ludu JS, Hare RF, Nix EB, et al. (2011) The biochemical properties of the Francisella Pathogenicity Island (FPI)-encoded proteins, IglA, IglB, IglC, PdpB and DotU, suggest roles in type VI secretion. Microbiology.

3. Fulop M, Manchee R, Titball R (1996) Role of two outer membrane antigens in the induction of protective immunity against Francisella tularensis strains of different virulence. FEMS Immunology & Medical Microbiology 13: 245-247.

4. Hickey AJ, Hazlett KRO, Kirimanjeswara GS, Metzger DW (2011) Identification of Francisella tularensis outer membrane protein A (FopA) as a protective antigen for tularemia. Vaccine.

5. Sjostedt A, Kuoppa K, Johansson T, Sandstrom G (1992) The 17 kDa lipoprotein and encoding gene of Francisella tularensis LVS are conserved in strains of Francisella tularensis. Microbial pathogenesis 13: 243-249.

6. Ludu JS, De Bruin OM, Duplantis BN, Schmerk CL, Chou AY, et al. (2008) The Francisella pathogenicity island protein PdpD is required for full virulence and associates with homologues of the type VI secretion system. Journal of bacteriology 190: 4584.

7. Nano FE, Zhang N, Cowley SC, Klose KE, Cheung KKM, et al. (2004) A Francisella tularensis pathogenicity island required for intramacrophage growth. Journal of bacteriology 186: 6430.

8. Mohapatra NP, Balagopal A, Soni S, Schlesinger LS, Gunn JS (2007) AcpA is a Francisella acid phosphatase that affects intramacrophage survival and virulence. Infection and immunity 75: 390.

9. Child R, Wehrly TD, Rockx-Brouwer D, Dorward DW, Celli J (2010) Acid phosphatases do not contribute to the pathogenesis of type A Francisella tularensis. Infection and immunity 78: 59-67.

10. Hager AJ, Bolton DL, Pelletier MR, Brittnacher MJ, Gallagher LA, et al. (2006) Type IV pili mediated secretion modulates Francisella virulence. Molecular microbiology 62: 227-237.

11. Buchan BW, McCaffrey RL, Lindemann SR, Allen L-AH, Jones BD (2009) Identification of migR, a regulatory element of the Francisella tularensis live vaccine strain iglABCD virulence operon required for normal replication and trafficking in macrophages. Infection and Immunity. pp. 2517-2529.
