# Supplementary material for: Rapid Focused Sequencing: A Multiplexed Assay for Simultaneous Detection and Strain Typing of Bacillus anthracis, Francisella tularensis, and Yersinia pestis
Source: PLoS One. 2013 Feb 13;8(2):e56093. doi: 10.1371/journal.pone.0056093 (PMC3572037; doi:10.1371/journal.pone.0056093)
Supplement: Table S3 — Yp target description, primer sequences, and 5′ fluorescent labels. Positions of amplicon boundaries based on bv. orientalis CO92 chromosome, pMT, pPCP; Genbank Acc# AL590842.1, AL117211.1, AL109969.1, respectively, and amplicon lengths based on in silico range observed in Yp whole genome strains are also noted. (DOCX) [file pone.0056093.s009.docx]

**Table S3***.* ***Yp* target description, primer sequences, and 5’ fluorescent labels.**

| *Yp* Targets | Position  ori CO92 | Forward Primer/Reverse Primer | Size [bp] | Gene Category: Function |
| --- | --- | --- | --- | --- |
| *glg*X | 4,429,378-4,429,034 | TMR-TTTGACTTAGGCACGGTACTGG/ ATCCAGCAAGGTGAAACCATCA | 345 | Metabolism: glycogen debranching enzyme/α-amylase involved in biodegradative metabolism. May be involved in low temperature growth adaptation [[1](#_ENREF_1)] |
| *glp*D | 4,421,230- 4,421,665 | TMR-CGCTGTTTCGAACATTCAGAGG/ GGACTTCACCGCCGTGTTTA | 436 and  529 | Metabolism: glycerol-3-phosphate dehydrogenase, essential enzyme in glycerol metabolism pathway. Deletion inactivates gene in bv. *orientalis* strains [[2](#_ENREF_2)] |
| *ail* | 3,248,000- 3,248,433 | ROX-TAAAGCCCCAGCTATAAGGCCA/ GACTGGGGAGTGATAGGTTCAT | 435-438 | Virulence: attachment inversion locus; part of *omp* locus. Mediates binding to host fibronectin and Mediates binding and Yop delivery [[3](#_ENREF_3), [4](#_ENREF_4)]. |
| *nuo*G | 2,863,082- 2,862,673 | FAM-GACTGGACGCAACTGGATC/ GCCGGTATGGAATCGAAGTAAT | 409-410 | Metabolism: NADH dehydrogenase subunit G; part of *nuo* operon. |
| YPO1976 | 2,245,631- 2,246,077 | GAGCAGATAAATGTTGACTATCTATC/ FAM-TGTGCTAATGACAACTAACTGG | 441-477 | Metabolism: conserved protein with anthranilate synthase/aminase component homology. Contains a VNTR repeat near the 5’ end. |
| *arg*S-YPO2047 | 2,325,048- 2,325,471 | TMR-CTCGTCGAGTGTTGACAGCAAG/ TCAATGATCTCTTCCAGGCCGT | 424-448 | Protein Biosynthesis: intergenic sequence between arginyl-tRNA synthase (*arg*S) and YPO2047 (conserved metal-binding protein). Contains a VNTR repeat |
| pMT_*ymt* | 74,477- 74,808 | ATTGGAAACTACTTTCATCATCTAAG/ FAM-GCAGCAGATAACCCATTCATAA | 331-332 | Virulence: murine toxin/phospholipase D encoded on pMT1; required for survival in blood plasma[[5](#_ENREF_5)]. |
| pMT_*caf*1 | 85,975- 86,391 | CCATTGCATTATTTGGAACTATTGC/ FAM-CAATTGAGCGAACAAAGAAATCC | 417 | Structure, Virulence: F1 capsule antigen, a major protein of the F1 capsule encoded on pMT1 [[6](#_ENREF_6), [7](#_ENREF_7)] |
| pPCP_*pla* | 7,135- 7,603 | TMR-ACGTTTCAGTTGGACAGCTACA/ TCAGAAGCGATATTGCAGACCC | 469 | Virulence: plasminogen activator Pla, a plasminogen-activating protease, essential for cause of primary pneumonic plague (deep tissue dissemination) [[8](#_ENREF_8)] |
| pPCP_*pst-*PCP06 | 5,682- 6,078 | ACTGTCTTTCTCCATCTCCGTA/ FAM-CTGTTTTTCTGCTGCGAATCTT | 395-398 | Resistance: intergenic sequence between pPCP-encoded Pesticin gene (antibacterial protein) and ORF PCP06. |

Positions of amplicon boundaries based on bv. *orientalis* CO92 chromosome, pMT, pPCP; Genbank Acc# AL590842.1, AL117211.1, AL109969.1, respectively, and amplicon lengths based on *in silico* range observed in *Yp* whole genome strains are also noted.

**References**

1. Bresolin G, Neuhaus K, Scherer S, Fuchs TM (2006) Transcriptional analysis of long-term adaptation of Yersinia enterocolitica to low-temperature growth. Journal of bacteriology 188: 2945.

2. Motin VL, Georgescu AM, Elliott JM, Hu P, Worsham PL, et al. (2002) Genetic variability of Yersinia pestis isolates as predicted by PCR-based IS100 genotyping and analysis of structural genes encoding glycerol-3-phosphate dehydrogenase (glpD). Journal of bacteriology 184: 1019.

3. Felek S, Krukonis ES (2009) The Yersinia pestis Ail protein mediates binding and Yop delivery to host cells required for plague virulence. Infection and immunity 77: 825.

4. Tsang TM, Felek S, Krukonis ES (2010) Ail binding to fibronectin facilitates Yersinia pestis binding to host cells and Yop delivery. Infection and immunity 78: 3358.

5. Hinnebusch BJ, Rudolph AE, Cherepanov P, Dixon JE, Schwan TG, et al. (2002) Role of Yersinia murine toxin in survival of Yersinia pestis in the midgut of the flea vector. Science 296: 733.

6. Cavanaugh DC, Randall R (1959) The role of multiplication of Pasteurella pestis in mononuclear phagocytes in the pathogenesis of flea-borne plague. The Journal of Immunology 83: 348.

7. Cowan C, Jones HA, Kaya YH, Perry RD, Straley SC (2000) Invasion of epithelial cells by Yersinia pestis: evidence for a Y. pestis-specific invasin. Infection and immunity 68: 4523.

8. Lathem WW, Price PA, Miller VL, Goldman WE (2007) A plasminogen-activating protease specifically controls the development of primary pneumonic plague. Science 315: 509-513.
